# Intermittent Motion in Desert Locusts: Behavioural Complexity in Simple Environments

**DOI:** 10.1371/journal.pcbi.1002498

**Published:** 2012-05-10

**Authors:** Sepideh Bazazi, Frederic Bartumeus, Joseph J. Hale, Iain D. Couzin

**Affiliations:** 1 Department of Zoology, University of Oxford, Oxford, United Kingdom; 2 Center for Advanced Studies of Blanes CEAB-CSIC, Girona, Spain; 3 Department of Ecology and Evolutionary Biology, Princeton University, Princeton, New Jersey, United States of America; University of Guelph, Canada

## Abstract

Animals can exhibit complex movement patterns that may be the result of interactions with their environment or may be directly the mechanism by which their behaviour is governed. In order to understand the drivers of these patterns we examine the movement behaviour of individual desert locusts in a homogenous experimental arena with minimal external cues. Locust motion is intermittent and we reveal that as pauses become longer, the probability that a locust changes direction from its previous direction of travel increases. Long pauses (of greater than 100 s) can be considered reorientation bouts, while shorter pauses (of less than 6 s) appear to act as periods of resting between displacements. We observe power-law behaviour in the distribution of move and pause lengths of over 1.5 orders of magnitude. While Lévy features do exist, locusts' movement patterns are more fully described by considering moves, pauses and turns in combination. Further analysis reveals that these combinations give rise to two behavioural modes that are organized in time: local search behaviour (long exploratory pauses with short moves) and relocation behaviour (long displacement moves with shorter resting pauses). These findings offer a new perspective on how complex animal movement patterns emerge in nature.

## Author Summary

The movement of organisms is an essential feature of life and is fundamental to almost all ecological and evolutionary processes. The motion of animals can have a significant impact on the environment, for example on the distribution of resources, habitat fragmentation or the spread of pests and diseases. Locusts exhibit one of the most devastating examples of animal movement, where locust swarms are a significant global pest. Therefore identifying the mechanisms of such movements is critical in understanding a range of ecological processes. An important challenge in studying animal motion is identifying the drivers of the complex movement patterns generated by organisms. Movement patterns may be the result of interactions between animals and their environment or may be directly the mechanism by which their behaviour is governed. Here we examine the movement behaviour of individual desert locusts in a homogenous experimental arena with minimal external cues. These findings offer a new perspective on how complex animal movement patterns emerge in nature.

## Introduction

An essential focus of experimental and theoretical studies of animal movement is to reveal the underlying drivers (internal and external) of the complex statistical patterns of animal motion that appear in nature [1]. Such patterns can be considered to emerge as a result of interactions between organisms and their environment [1], or they may be directly the mechanism by which behavioural processes are governed [2], [3], [4]. Examining how animals move and the properties of their movement at different scales is critical in understanding the drivers of the complex patterns found in animal movement, and one of the main goals of ecological research [5].

Observations of animal locomotion have shown that intermittent movements are a common feature [6], [7], [8]. Movement is not constant or continuous but rather intrinsically discrete, interrupted by accelerations, decelerations or pauses [9]. Intermittent locomotion can be found in terrestrial, aquatic and aerial environments and occur in a range of ecological contexts, such as search behaviour, habitat assessment or the pursuit of prey [6]. It implies that animals can discretize their movement behaviourally in a series of move lengths, pauses, and turns in response to certain cues of the changing environment [2], [6], [8], [10]. Behavioural intermittence may perhaps be due to an animal's energetic restrictions, to allow an animal to recover from fatigue, for prey detection [6], or for navigation, such as path integration [11].

Interruptions to continuous motion are also thought to be adaptive in search processes, resulting in increased search efficiency [9], [10], [12]. They can facilitate sharp reorientations that may break the animal persistence of its previous directional motion and, depending on the temporal pattern, can thereby allow it to explore effectively an area [2], [9], [10]. Such a process may be beneficial to animals living in dynamic and fluctuating environments, where situations are likely to change as time progresses [13]. This idea that interruptions might be adaptive by enhancing behavioural plasticity is not new [14], but is yet to be explored empirically for animal movement behaviour. In addition, if there is alternation of scanning and non-scanning phases, the search process itself becomes intermittent. Theoretical models have shown that random searches with optimal proportions of scanning/non-scanning phases enhance encounter success [15], [16], [17].

Behavioural intermittence appears as an essential characteristic of the movement patterns exhibited by many animals [8] and has long since been documented [18], and detailed experiments on intermittency are becoming increasingly common [7], [19], [20], [21], [22], [23], [24], [25]. Therefore there is limited knowledge about the causes of movement: is an organism's motion internally governed or a reflection of their external environment? Thus far, few studies have examined long-term animal motion under limited external cues and there is a limited understanding of the null movement patterns of motion without contributing external influences [26], [27].

One of the greatest challenges of movement ecology is linking the statistical properties of movement to specific behaviours [28] and identifying behavioural transitions in the movement patterns. To achieve this, an elemental view of the movement path is needed, with identification of all displacements and pauses in a trajectory and associating these to the behaviour of the organism [1]. Getz and Saltz (2008) suggested identifying the potential determinants of movement using canonical activity modes (CAMs) consisting of shorter duration fundamental movement elements (FMEs) [29]. Behavioural modes have previously been identified in elk, defined by relocation distances and turning behaviour [30], and switching between different behavioural modes has been observed at various spatiotemporal scales [31]. Such studies highlight the importance of examining high temporal resolution data over different scales in order to identify the mechanistic determinants of movement.

Previous ecological studies often involve short length (spatial or temporal) empirical data, which makes it difficult to assess the statistical properties of movement behaviour [32], [33], [34], [35] or, more recently, contain high resolution long length movement data of animals in their natural environment but under uncontrolled conditions [19], [22], [24], [25], [36], [37], [38]. Unravelling the drivers of the complex statistical patterns (including intermittence) observed in animal movement requires high resolution data on animal movement over large spatiotemporal scales [4], [12], and controlled conditions (for example, in the absence of strong environmental fluctuations or interactions with other individuals).

Here we examine the movement of isolated individual juvenile desert locusts, *Schistocerca gregaria*, in a homogeneous experimental arena, thus minimizing environmental fluctuations that may influence motion. Desert locusts are typically found in relatively barren land where the location of resources may be scarce and/or unpredictable, hence, the experiment depicts a common ecological situation of the species. We record locusts' movements by locating them at a fine temporal resolution (every 0.2 s) for 8 h. Under such simple conditions, we consider in detail the nature of behavioural intermittency and provide a comprehensive view of the complex structure and the long-term variability of the intermittent patterns observed in locusts. We quantify the role of pauses as a turning (reorientation) mechanism, and we examine the distribution of move and pause lengths. We also quantify short and long-term correlation properties of moves and pauses, unveiling the overall organization of move and pause sequences. The analysis allows us to determine the relationship between the key features of locusts' movement: moves, pauses and reorientations, and therefore to understand how complex search patterns are generated by organisms under minimal external sensory stimuli.

## Materials and Methods

### Experiments

Healthy, intact freshly moulted gregarious desert locusts (*Schistocerca gregaria*) in the 5^th^ (final nymphal) instar, reared under conditions described in Roessingh *et al.* [39], were placed in groups of 20 individuals per plastic cage (30×20×10 cm), each with a mesh roof, containing sawdust, an expanded aluminium perch and a water supply. These were fed one of three dry, granular synthetic diets *ad libitum* for 48 h, as described in [40]. We found no significant differences among diets on the frequency distribution of moves and pauses (comparing the power-law scaling exponent, μ among diets; ANOVA: F_(2,90)_ = 0.041, p = 0.959, data were log transformed to achieve normality; and ANOVA: F_(2,90)_ = 1.906, p = 0.154, respectively). Furthermore, Bazazi *et al.* (2011) previously found that nutritional state has minimal influence on the proportion of time spent moving and on the speed of isolated locusts. Marching and feeding behaviour are low and irregular 24 h post moult [18] but by 48 h locusts have high and uniform marching and maintain a high food intake.

After 48 h a single locust was placed in a ring-shaped experimental arena (80 cm diameter, walls 52.5 cm high and a central dome 35 cm diameter [41]). 40 W fluorescent lamps illuminate the arena and reduce visual stimuli available to locusts above the arena. This setup effectively simulates a large featureless environment within a reasonable space both for experimental tractability and for the purposes of tracking, which has inherent restrictions due to resolution constraints. The motion of the locusts in the arena was then filmed for 8 h using a digital video camera (Canon XM2). Automated digital tracking software [41], [42], which captured images at a rate of 5 times per s, was used to analyse the video footage and obtain information regarding the position, speed and direction of an individual between successive frames. Each trial was started in the morning between 9:00AM–10:00AM. We carried out a total of 93 experimental trials (93 individuals). A video clip of an experimental trial is available in Supporting Information (Video S1). No individual was used more than once.

### Data analysis

#### Intermittent movement: moves and pauses

**Figure 1:**
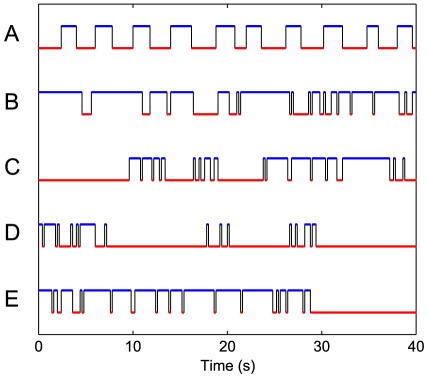
The intermittent nature of movement. Individual motion can be discretized into a series of move, blue, and pause, red, lengths. The black lines indicate switches between these states. The pattern of movement is shown for an individual with Brownian motion (**A**) and for individual locusts observed in experiments (**B–E**) for 40 s. We calculated a total of 44,710 move lengths and 60,103 pause lengths for all individuals. Since our measurements of locusts' movements were recorded per frame, we treated move and pause length durations as pre-binned (discrete) data, rather than continuous (following Edwards *et al.* [68]).

The observed motion of individual locusts was made up of moves and pausing bouts of variable length (see Figure 1). Thus the motion of individuals can be discretized into a series of moves and pauses with “moving” defined as displacement greater than 0.3 cm between successive frames (0.2 s) and a pause as displacement less than or equal to 0.3 cm (during which a locust can show resting or fidgeting behaviour [43]). The threshold for moving was calculated by plotting histograms of locusts' speeds between successive frames and selecting the speed just below the second peak in the distribution (the first peak was at speed = 0). This threshold is similar to that used in Bazazi *et al.* [40], [41] and Buhl *et al.* [42]. Using these criteria we determined whether a locust was moving in each frame, and therefore the duration of moves and pausing bouts. Data from individuals that were found within 3 cm of the outer wall and central dome were excluded from the analysis to remove edge effects (analysis with the inclusion of data from individuals found within 3 cm of the arena wall show similar qualitative results for the distribution of move and pause lengths and suggest that move length truncation might be an intrinsic property of locusts' movement rather than simply an artefact of the experimental design- see Figure S1).

**Figure 2:**
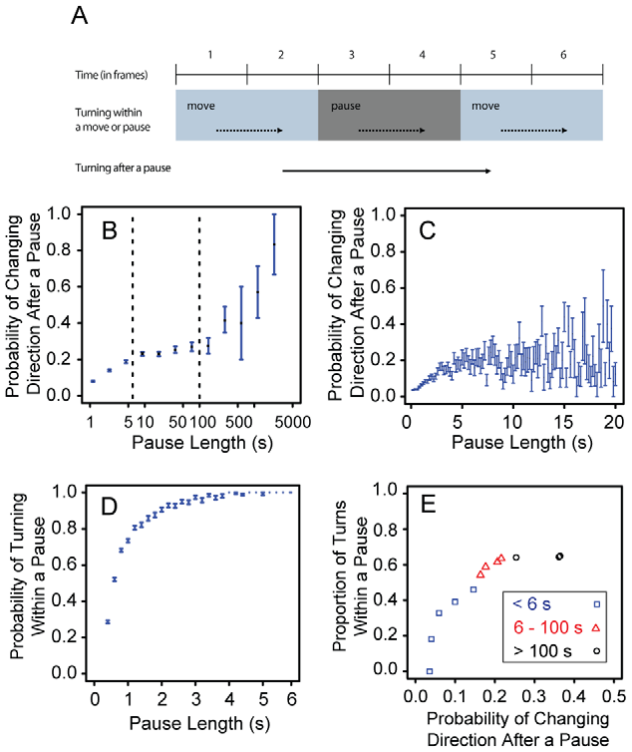
Individual behaviour after and within a pause. (**A**) showing our calculation of locust turning behaviour within moves or pauses, or after a pause. We define a turn as a change from CW to ACW movement or vice versa. Arrows indicate the time steps for which the switch between CW to ACW was considered. Within a move or pause only consecutive time steps were examined (dotted arrows). For turning after a pause, the time steps immediately before and after the pause were considered (solid arrow). (**B**) shows the mean probability of changing direction after a pause for observed pause lengths (s), using log-binned averages. The left and right dashed lines show 6 s and 100 s, respectively. (**C**) shows the mean probability of changing direction after a pause for pause lengths of up to 20 s on a normal scale. (**D**) shows the mean probability of turning within a pause for different pause lengths. We have presented pause lengths up to 6 s as pause lengths greater than 6 s show a probability of one. For (**B–D**) error bars show 95% confidence intervals of the mean. (**E**) shows the relationship between the mean proportion of turns within a pause and the probability of changing direction after a pause for pause lengths of: less than 6 s (blue squares); between 6 s and 100 s (red triangles); and greater than 100 s (black circles). Each data point is a mean calculated from data within logged bin classes for pause length.

In order to understand the behaviour of an individual during pauses, we examined the number of changes in direction during a pause for all pauses, for all locusts. Our experimental setup, consisting of a circular arena and central dome, meant locusts were able to move continuously around the arena, and allowed us to reduce the system to a one dimensional representation of locust movement (as in Buhl *et al.* [42]). Thus we determined whether a locust showed a change in direction (turn) by examining whether it switched its head direction from clockwise (CW) to anti-clockwise (ACW) or vice versa between time steps. To do this we calculated the change in the sign of the cross product of its positions between successive frames. Thus we examined whether or not there had been a change of direction, from CW-ACW movement, rather than measuring the turning angle. We defined a turn as a change from CW to ACW movement or vice versa. We quantified the CW-ACW switching behaviour within pauses and moves (see also Figure 2A) and the proportion of CW-ACW switches within a pause/move. This information allowed us to compare the probability of turning and the proportion of turns within moves and pauses, and therefore determine whether a pause can be considered a reorientation bout. Furthermore we determined whether there had been a change from CW to ACW movement or vice versa between the time steps immediately before and after a pause in order to see how the duration of a pause affects this probability.

#### Behavioural mode analysis

In order to understand the relationship between moves and pauses, we carried out correlation analyses for move lengths, pause lengths and between moves and pauses (see Text S1 for details). In addition we classified local search and relocation behavioural modes based on a partial sums (PS) approach [44]. The PS algorithm is a form of the Cumulative Sum Analysis [45], widely used in many disciplines (e.g. industrial engineering, economics, and medicine) to analyse the deviations of a process from a target or reference value. This method uses a cumulative sum equation to generate a sequence of observations (time series), which is then analysed to identify the main transitions between different phases/modes/regimes in the variable of interest. The PS algorithm can allocate sequences of moves and pauses into two behavioural modes: i) local search, i.e. sequences of long pauses and short moves, and ii) relocations, i.e. sequences of long moves and short (non-turning) pauses.

We have adapted Knell and Codling (2011)'s algorithm [44] in the following way. For each experiment, we modified the move and pause length time series by assigning negative signs to pauses and positive signs to moves. We used the value T = 0 as the reference value in order to unambiguously distinguish the contribution of moves and pauses to the cumulative sum equation [44], [45]. In the PS algorithm one assumes minimum time duration for a behavioural mode to exist, i.e., the time threshold parameter (ε). The latter allows the allocation of breakpoints in the cumulative time series, distinguishing distinct modes. We chose ε = 5 minutes (other threshold values were also tested but did not qualitatively change the results- see Figure S2). In addition, we also identify fast and slow moves by measuring the angular speed, a measure of how fast the locust moves around the arena (see Text S1 for details).

#### Detection of power-law distributions

In order to understand the statistical properties of locusts' movement we examined the frequency distribution of move lengths and pausing bout lengths for each locust. We fitted several simple probabilistic models, often observed in dispersal or movement data [46], [47] to our data: a bounded (truncated) power-law model, a pure power-law model, and a bounded exponential model. A mathematical description of these models can be found in Text S1. In order to determine which probabilistic model best fits the distribution of moves and pauses for each locust, we carried out sequential pointwise model comparison (SPWMC) tests. The SPWMC analysis consisted of conducting point-wise maximum likelihood estimates, and based on Akaike weight computations (wAIC) examining the relative likelihood of each model compared with the likelihood of the best-fit model [36]. The value of the wAIC gives the weight of evidence in favour of a model, where wAIC = 1 is the maximum weight of evidence. The analysis also explored whether different models could fit different regimes of the data.

In order to determine accurately the scaling exponent μ of the bounded power-law behaviour observed in the data, we used maximum likelihood techniques and fitted two general models consisting of: (i) a power-law model with a stretched exponential function for the tail (i.e. large moves/pause lengths), and [48] the same model as (i) but including an exponential distribution for the small moves/pause lengths (for more details on the models see Text S1). Despite some variability at shorter moves/pause lengths, which show both power-law and exponential variability, most of the individual locusts behaved similarly in statistical terms above certain move/pause length values. A power-law model with a stretched exponential tail function could be well fitted to data from all individuals (for individual locust data analyses see Text S1, Figure S3, Figure S4, Table S1, Table S2). The stretched exponential distribution is an exponential distribution with a parameter, β (where 0<β<1), which accounts for deviations from exponential behaviour at the tail (β = 1 represents pure exponential behaviour, and the smaller the β value, the fatter the tail). We pooled the data for all locusts together to get more statistical power on our analysis. We computed the distributions of move/pause lengths to represent the behaviour of an “average” locust and fitted a power-law with a stretched exponential tail model to these data.

We computed the empirical complementary cumulative distribution functions (CCDFs) by plotting for a variable *x* (here either move or pause lengths in seconds) the proportion of observations that were equal to or larger than *x*, i.e., *P(X≥x)* on a logarithmic scale [38], [49]. We also computed the empirical probability density functions (PDFs) for the move and pause lengths (see Text S1 for calculations). We excluded pause lengths greater than 1000 s (which account for 0.02% of all pause lengths) to remove the effects of those locusts considered to be exhibiting atypical behaviour. Once we performed a fit of our model to the empirical data, we carried out model criticism on our analysis by visually examining how our observed distributions deviate from the expected distribution +/−2 SD [38], [50].

## Results

### Pauses as reorientation bouts or rests

Our quantification of locusts' turning behaviour both within pauses and within moves demonstrates that a change in direction is more likely to be found in a pause than during a move. The mean proportion of pauses with changes in direction from total bouts (moves and pauses) with changes in direction is 0.8609 (+/−0.0966, one SD). By contrast the mean proportion of moves with changes in direction is 0.098 (+/−0.0726, one SD). Therefore we can consider moves as displacements without reorientations, and pauses as opportunities for turns.

We then considered whether the duration of the pause influences the mean probability that a locust changes direction after a pause (Figure 2B). This probability shows a strong positive relationship with pause length for pauses lasting up to 6 s (Figure 2B and Figure 2C). For pauses between 6 s and 100 s, very little correlation appears with the probability of changing direction, remaining between 0.2 and 0.3 (Figure 2B). Increasing pause length beyond 100 s results in a further increase in the mean probability of changing direction (Figure 2B). Our data also show that the mean probability of turning within a pause, reflecting the fidgeting behaviour of locusts, increases as the pause duration increases, and plateaus to one at 6 s (Figure 2D).

The mean proportion of turns within a pause is significantly higher for pause lengths of 100 s or greater (0.6505+/−0.0191, one SD) than for pause lengths between 6 s and 100 s (0.5771+/−0.1049, one SD; T-test: p<0.0001, T-statistic = −10.7966, Df = 4830). There also exists a positive relationship between turning behaviour within a pause and the probability of changing direction after a pause (Figure 2E) for pauses shorter than 100 s. For pauses longer than 100 s the probability of turning within a pause remains just above 0.6, and does not affect the probability of changing direction after the pause. Therefore increasing turning within a pause, that is, increasing fidgeting while paused, increases the likelihood that after the pause the locust changes direction, but only for pauses lasting less than 100 s (Figure 2E).

We also calculated the most influential pause length for changes in direction after a pause, which emerges from the combination of the distribution of pause lengths and the probability of changing direction for a given pause length (Figure S5). We find that even though the probability of turning is very low in the smaller pause lengths, the latter contribute more to turning behaviour, when considering the overall locust trajectory motion, because they are overwhelmingly abundant.

### Detecting power-laws in move and pause length distributions

**Figure 3:**
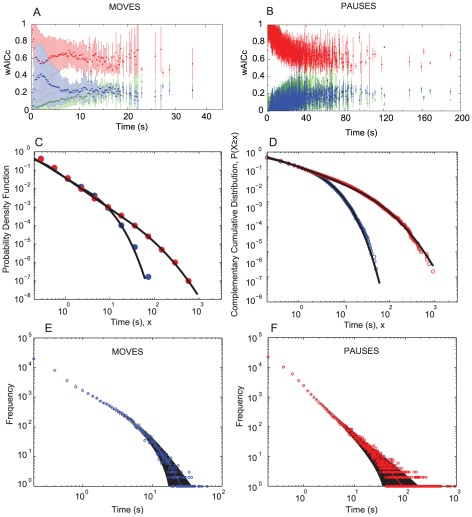
Detailed analyses of moves and pauses. The SPWMC analysis with bounded power-law, red, pure power-law, green, and bounded exponential, blue, for moves (**A**) and pauses (**B**). A value of weighted Akaike information criteria (wAIC) of one gives the maximum weight of evidence in favour of the models. The results here are means for all individuals. Error bars indicate +/− one SD. The probability density functions (**C**) and the complementary cumulative distribution plots (**D**) and for moves, blue, and pauses, red, showing the empirical data and the model fits for the power law with a stretched exponential tail model (black line). μ is the scaling parameter of the power-law, β is a parameter that tells us the deviation of the tail from an exponential. For moves: μ = 1.49, β = 0.55; for pauses: μ = 1.67, β = 0.23. (**E–F**) shows the observed and expected distributions for moves and pauses. Log-log plot of the frequency distribution of different move (**E**) and pause lengths (**F**). Open circles show the observed distribution from our data and dots show the expected distribution from the model fit (a power-law with a stretched exponential tail model). We assume a Poisson distribution for the deviations from the expected values for each bin. The black error bars show +/−2 SD from our expected value.

The results of the SPWMC tests for the moves and pauses averaged for all individuals are shown in Figure 3A–B. For shorter moves we see an overlap between the exponential and bounded power-law models. This indicates that shorter moves follow a mixture of probabilistic models. However for longer moves (the tails), the bounded power-law model is more dominant (Figure 3A). The dominance of the bounded power-law model in Figure 3B strongly favours a very heterogeneous (fat-tailed) distribution of pauses. We note that SPWMC tests do not show actual fits, but instead are meant to be a first explorative analysis to compare among a reasonable set of models.

Figure 3C–D shows the CCDFs and the PDFs, respectively, for the moves and pauses once the data have been pooled (for individual locust analyses results see Text S1, Figure S3, Figure S4, Table S1, Table S2). The value of the Lévy exponent μ in the negative power-law equation fitted to the data is 1.49 for moves and 1.67 for pauses. For moves, θ, the cut-off where the stretched exponential tail begins (also determined from the model fit), is 8 s, and for pauses θ is 15.27 s.

Figure 3E–F also allows us to check visually how well our model fits our observed data. We find that most deviations exist at the beginning and at the tail of the move and pauses length distributions, with pauses showing larger deviations. However the deviations are small and do not show a systematic pattern. Error accumulations in the smallest and largest regimes of our variables are responsible for the spurious results when using standard goodness-of-fit tests (see Text S1 for further details). However, Figure 3 demonstrates that our model (a power-law with a stretched exponential tail) provides a reasonably good fit to the data for moves and pause distributions.

### Defining behavioural modes: combining moves and pauses

**Figure 4:**
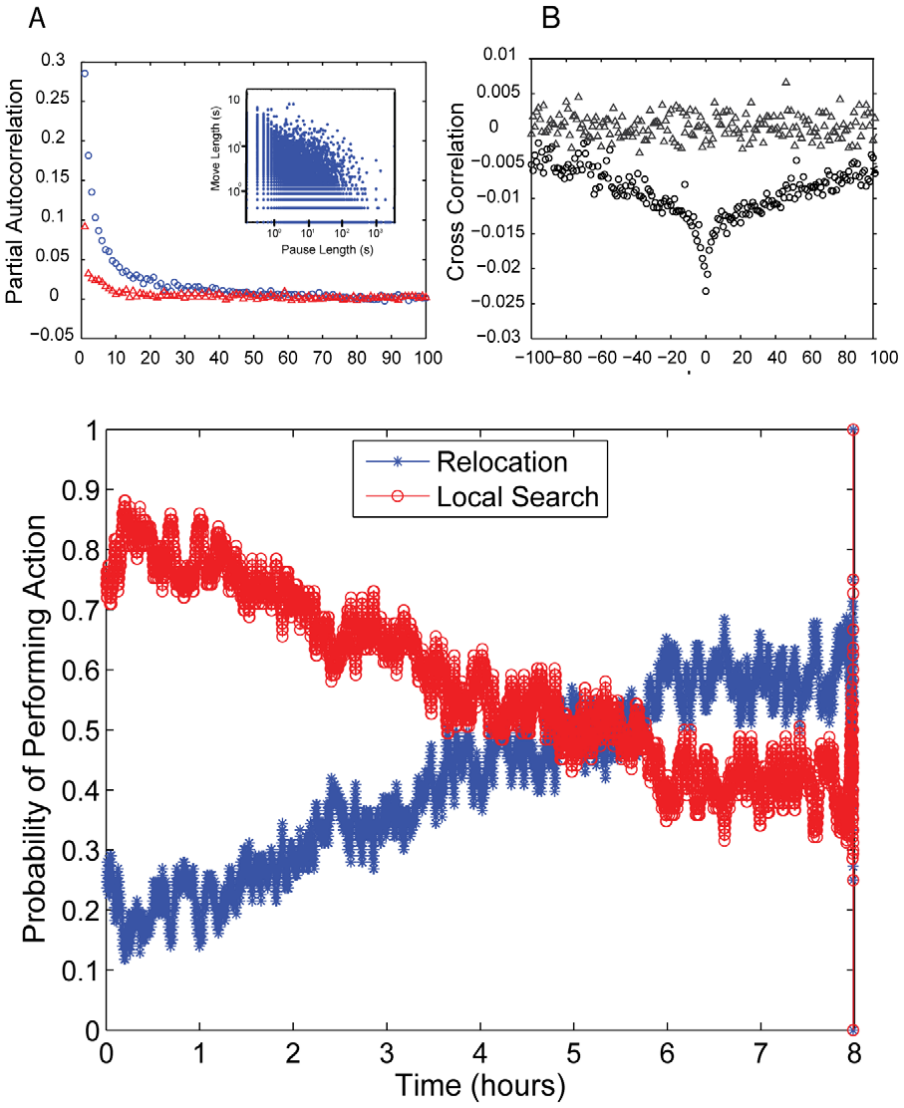
Correlation analysis for moves and pauses and behavioural modes. Partial autocorrelation analysis results (**A**) for moves, blue circles, and pauses, red triangles, reveals that moves are positively correlated and pauses show a slight positive, if any, correlation, therefore moves show much stronger local correlations than pauses. Inset shows first order correlation between moves and pauses (see Figure S6). A cross correlation of moves and pauses (**B**) shows a high negative correlation at a local scale, circles, which persists for larger time scales. The shuffled data are also shown (triangles) for comparison. (**C**) shows the probability that a locust performs two different behaviours (local search and relocation) over the course of the 8 hr experiment. Relocation behaviour (blue asterisks) is defined as long moves preceded or followed by short pauses. Local search behaviour (red circles) is defined as long pauses preceded or followed by short moves. Behavioural modes are classified using the Partial Sums algorithm with a minimum time threshold of 5 min.

When we examined the relationship between moves and pauses we observed strong, negative, first-order correlations (Figure 4A inset and Figure S6). Short pauses are associated with moves of all lengths. Longer pauses however are more likely to be associated with shorter moves. Furthermore locusts tend not to exhibit large move lengths and large pauses together. We therefore carried out more complete correlation analyses on moves and pauses. Our partial autocorrelation results reveal that moves show much stronger local correlations than pauses (Figure 4A). Cross-correlation analysis between moves and pauses reinforce the idea that there is a negative correlation, particularly at local scales (Figure 4B). Thus long moves tend to be associated with short pauses and vice versa. In addition, long moves account for faster, and more energetic, circling in the arena. Thus, non-turning short pauses could allow for some energy recovery. In particular we observed that the average angular speed increased as move lengths increased, reaching a saturation of 15 degrees/s, at moves of 15 s in duration (see Figure S7).

For each experiment, the PS algorithm allocates sequences of moves and pauses into two behavioural modes: i) local search, i.e. sequences of long pauses and short moves, and ii) relocations, i.e. sequences of long moves and short (resting and non-turning) pauses. At the population-level, that is, averaging over all the individuals, we can obtain the probability that a locust is in one mode or another over time. The data show that the probability that a locust performs relocation behaviour at the beginning of the experiment is small (0.2) but increases as the experiment progresses to approximately 0.6. Conversely a locust performs local search at the beginning of the experiment at a probability fluctuating around 0.8 within the first 1.5 hrs but this decreases as the experiment progresses. After almost 5 hours there is a shift and both modes stabilize around 0.5, with the probability of relocation being slightly larger than the probability of local search.

## Discussion

We have carried out a thorough statistical description of isolated locusts' motion using a large data set (93 experimental trials, each lasting 8 h, with positions and orientations acquired every 0.2 s). Controlled laboratory conditions were used to study movement, pausing and turning behaviours, thereby minimizing the amount of interference from external cues to individual motion. This is not to neglect the influence of environmental factors, but rather to help elucidate whether complex statistical properties of movement may be generated in their absence. Our results show that intermittence with Lévy signatures can be considered the behavioural template for search in individual locusts. It is however also clear from our analysis that intermittent patterns are constrained, perhaps by biomechanical, physiological or neurobiological factors, and in addition, can be modulated by the animal's internal state.

Our examination of turning behaviour during pauses suggests that pauses serve different functions, depending on their duration. Longer pauses (beyond 100 s) appear to act as reorientation bouts, which may serve to interrupt the persistence of previous directional motion since they are most likely to result in a complete change in direction of movement after the pause (Figure 2B). In addition longer pauses involve more turning within the pause itself, resulting from body rotation without displacement (Figure 2D–E). Such fidgeting behaviour could perhaps be the stochastic effect resulting from a locust scanning (and barely moving from) its surroundings. As locusts use optic flow to gather information from their environment, they are able to “see” their surroundings both when in motion and during fidgeting behaviour that involves head movements [51]. The shortest pauses (of less than 6 s) appear to act as periods of resting between displacements, as they do not involve a high probability of changing direction after the pause (Figure 2B–C) or greater turning within the pause (Figure 2D–E), and are associated with moves of variable length (Figure S6).

Our correlation analyses reveal that move and pause lengths are negatively correlated with one another (Figure 4B and Figure S6). The correlations of short (slow)/long (fast) moves with long (reorientation)/short (resting) pauses lead to different relative proportions of local search and relocation behaviour, which are organized in time (Figure 4).

Local search is characteristic of ‘pottering’ behaviour, during which a locust moves then stops at intervals to test its environment with its palps and antennae, resulting in frequent changes in direction [18]. Relocation behaviour may be associated with ‘marching’ activity, consisting of continuous locomotion in a persistent direction [18], [52]. Our results suggest that the two behavioural modes identified are steadily decreasing (local search) or increasing (relocations) up to approximately 6 hours, after which both behavioural modes show stationary fluctuations. We acknowledge the statistical issue of the degree of independence in sequential time series data of this sort. However this is an inherent problem with all such analyses, including that of this work.

The motivation resulting in an increase in relocation activity towards the end of our experiments is not explicitly explored here. However previous studies on locusts have revealed that increased marching may be associated with hunger; as the amount of food in their gut decreases, marching activity increases [18], [40]. Locusts may be investing more time in local explorations at the start of the experiment. As the cumulative information of non-available resources becomes stronger, local search and relocations appear to happen with more similar proportions: a stationary exploratory behaviour seems to emerge.

In our analysis of the distribution of moving and pausing step-lengths we observe that the probability of very long moves or very long pauses is small but not negligible (far beyond the Gaussian tail expectation), and that locusts' movements are better described by means of a general class of random walks known as Lévy walks [53], [54]. Our results show that power-law behaviour is naturally bounded to some range of scales [7], [55], [56], meaning that the time over which an individual can move or pause in a single bout is limited, perhaps owing to some physical constraints or to some strategic advantage [38].

When we pool our data for all individuals together (to obtain the average), we observe power-law behaviour of over 1.5 orders of magnitude for moves and pauses after which there is a cut-off and the stretched exponential tail begins (Figure 3). These results suggest power-law behaviour with additional complexity. The scaling exponents obtained from our data (μ = 1.49 for moves, μ = 1.67 for pauses) lie within the range expected from a Lévy walk (1<μ≤3) [54]. We find that locust behaviour shows movement patterns that are not entirely Ballistic (with exponent of μ≈1). Ballistic motion is useful to a foraging animal if targets are homogeneously located far away with respect to the initial searching position. Lévy patterns with μ≈2 [57], [58], [59], [60] become optimal in patchy landscapes, where far away and nearby targets exist [9], [61]. Recent results show the impact of landscape heterogeneity in optimal random search strategies, and suggest that the over dispersed and highly heterogeneous nature of desert vegetation [62], [63] could have promoted intermittent motion within the Lévy range: 1<μ≤2 [61], which we observe here in locusts.

The presence of power-law regimes in empirical distributions of animal movement data has generated much debate [2], [4], [10], [49], [64], [65], [66], [67], [68], however, there is strong empirical evidence for power laws in animal movement within natural habitats [36], [37], [38], [69], [70] and under experimental conditions [26], [27]. Our results suggest that while these patterns may result from interactions with the environment, they can also be generated internally. However Lévy distributions do not fully characterize locusts' movements. The behavioural template of locusts in the absence of environmental cues results from the relationship between moving, pausing and turning and involves both some physical constraints and some higher-order movement structure. In our experiments, internal state behavioural modulation may exist in association with a “starvation/satiation state”, or a “present/absent food memory” [9], [71]. The switch from local search behaviour at the beginning of the experiment towards relocation behaviour may be due to the general effects of food deprivation, which is known to result in increased marching [18], [40], either as food memory is lost or starvation levels increase.

We may understand complex intermittence as the interweaving of different behavioural modes [26] that are likely to be constrained by species-specific physical and biological factors. For example, fidgeting is physically impossible at small pauses but is constant at large pauses and large moves need to be interspersed with small (resting but not turning) pauses so that locusts can make large scale-free displacements in random directions. Future experiments should be designed to determine whether such behavioural constraints are driven at the biomechanical, the physiological or the neurobiological level. The idea of a null scale-free (Lévy-like) behavioural template may be in concordance with neuronal activity patterns, which in desert locusts also show a Lévy distribution with an exponent of approximately 1.5 [72], [73].

Overall, our results add upon the random paradigm debate in movement ecology [28] on whether internal states or external stimuli drive behavioural variability. Our findings suggest that the complex intermittent patterns observed are mainly internally shaped and governed. Therefore spontaneous and/or internally driven variability should be considered in order to achieve a comprehensive understanding of animal motor reactions to the environment, which is the ultimate goal in the field of movement ecology.

## Supporting Information

Figure S1 **Complementary cumulative distribution functions including and excluding boundary data.** The complementary cumulative probability distribution plots for moves (**A**) and pauses (**B**). The plots show the empirical data either including, blue, or excluding, red, data from individuals within 3 cm of arena walls. As we define move lengths based on rotational direction (CW/ACW), contact with the border could be included in on our definition of move length. Nevertheless, such contact can introduce new behavioural components that we have avoided to include in our main analysis. Analysis with the inclusion of data from individuals found within 3 cm of the arena wall show similar qualitative results for the distribution of move lengths (**A**); the power-law spans over slightly longer time scales and the stretched exponential tail starts later (fitted parameters: μ = 1.33, θ = 14 s, β = 0.75). For the distribution of pauses (**B**), the inclusion of data from the borders results in the stretched exponential tail being less pronounced such that the power-law spans over much longer time scales (fitted parameters: μ = 1.45, θ = 89.9 s, β = 0.579).
(DOC)

Figure S2 **Behavioural mode thresholds.** The probability of being in local search or relocation modes over time for different time duration thresholds. In the PS algorithm used to detect the two behavioural modes, the time duration threshold parameter represents the minimum time threshold for a behavioural mode to be sustained in order to be considered different from the previous mode. It is a constant numerical value that allows the allocation of breakpoints in the cumulative deviation series (Knell and Codling 2011) thereby distinguishing distinct modes, according to this minimum behavioural mode time duration threshold. Time thresholds of 1 min (top panel), 5 min (middle panel) and 10 min (bottom panel) were tested, meaning that a mode should last at least 1, 5, or 10 minutes. All thresholds tested yield the same qualitative results.
(DOC)

Figure S3 **Complementary cumulative distribution functions for move lengths.** Each subplot shows a log-log plot of the complementary cumulative distribution function of the empirical data (blue) of different move lengths, (*x*) exhibited by each individual locust and the best model fit, either the exponential followed by power-law with exponential tail model fit (red) or the power-law with exponential tail model fit (black). Numbers in each subplot indicate individual number (1 to 93).
(DOC)

Figure S4 **Complementary cumulative distribution functions for pause lengths.** Each subplot shows a log-log plot of the complementary cumulative distribution function of the empirical data (blue) of different pause lengths (*x*) exhibited by each individual locust and the best model fit, either the exponential followed by power-law with exponential tail model fit (red) or the power-law with exponential tail model fit (black). Numbers in each subplot indicate individual number (1 to 93).
(DOC)

Figure S5 **The influence of pause length on the probability of turning after a pause.** Bar chart showing, on the left axis, the proportion of data points (white) and the mean probability of turning after a pause (black) for each pause length bin class (log binned classes). The influence (grey) of the data points on the mean probability of turning after a pause for each pause length is also shown (right axis). This was calculated by multiplying the mean probability of turning by the proportion of data points within each pause length bin class.
(DOC)

Figure S6 **Pause lengths and move lengths proceeding or following pauses.** The relationship between pause length (s) and move length (s) for moves immediately preceding (**A**) or following (**B**) pauses for all locusts. The relationship between pause length and the following move length, or pause length and the preceding move length show a similar pattern (since moves after one pause are before another).
(DOC)

Figure S7
**Angular speed for different move lengths.** The angular speed (in degrees per s), ω, is measured as dθ/dt, where dθ is the angle (in degrees) moved between the first and last frame of the move, and dt is the move length in s. The black line shows a non-linear least squares fit (of the type: 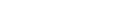, where *a* = 3; *c* = 18, in Matlab 2010b) to the data. The mean angular speed saturates to 15 degrees/s at move lengths of 15 s.(DOC)

Table S1 **Individual model fit results for moves.** For the distribution of moves of each individual locust we calculated: the best fit model to the data, either model 1 or model 2, Model; the total number of move lengths, N; the maximum move length, Max (in s), for each individual (the minimum move length for all individuals is 0.2 s); the Lévy exponent, μ; the parameter that tells us the deviation of the tail from an exponential, β (where β = 1 is an exponential tail, and β = 0 is a power-law tail); for model 1 the mean lifetime (or characteristic) move length (in s), θ (1) ; for model 2 the move length value (in s) delimiting the beginning of the power law regime, x2(2); move length value (in s) delimiting the end of the power law regime, x3, (the dots in column x3 show where model 1 was a better fit than model2); the negative log-likelihood function, NegLogLik; the goodness of fit test statistic value, GOF; the p-value from a goodness-of-fit test telling us whether the model is reliable or not (from Edwards *et al.* (2007)), where p-values>0.1 are a good fit of the model to the data (highlighted).
(DOC)

Table S2 **Individual model fit results for pauses.** For the distribution of pauses of each individual locust we calculated: the best fit model to the data, either model 1 or model 2, Model; the total number of pause lengths, N; the maximum pause length, Max (in s), for each individual (the minimum pause length for all individuals is 0.2 s); the Lévy exponent, μ; the parameter that tells us the deviation of the tail from an exponential, β (where β = 1 is an exponential tail, and β = 0 is a power-law tail); for model 1 the mean lifetime (or characteristic) pause length (in s), θ (1); for model 2 the pause length value (in s) delimiting the beginning of the power law regime, x2(2); pause length value (in s) delimiting the end of the power law regime, x3, (the dots in column x3 show where model 1 was a better fit than model2); the negative log-likelihood function, NegLogLik; the goodness of fit test statistic value, GOF; the p-value from a goodness-of-fit test telling us whether the model is reliable or not (from Edwards *et al.* (2007)), where p-values>0.1 are a good fit of the model to the data (highlighted).
(DOC)

Text S1 **The supporting text provides further details of the materials and methods used.** These include correlation analyses between moves and pauses, the behavioural modes analysis, and the quantification of move speeds. In addition, the supporting text also shows the mathematical description of the probabilistic models used in SPWMC, details of individual locust data analyses, and calculations of the different models used in our investigation.
(DOC)

Video S1 **A video clip of an experimental trial showing the movements of a single locust in the experimental arena.**
(AVI)
