# Supplementary material for: Intermittent Motion in Desert Locusts: Behavioural Complexity in Simple Environments
Source: PLoS Comput Biol. 2012 May 10;8(5):e1002498. doi: 10.1371/journal.pcbi.1002498 (PMC3349720; doi:10.1371/journal.pcbi.1002498)
Supplement: Figure S1 — Complementary cumulative distribution functions including and excluding boundary data. The complementary cumulative probability distribution plots for moves (A) and pauses (B). The plots show the empirical data either including, blue, or excluding, red, data from individuals within 3 cm of arena walls. As we define move lengths based on rotational direction (CW/ACW), contact with the border could be included in on our definition of move length. Nevertheless, such contact can introduce new behavioural components that we have avoided to include in our main analysis. Analysis with the inclusion of data from individuals found within 3 cm of the arena wall show similar qualitative results for the distribution of move lengths (A); the power-law spans over slightly longer time scales and the stretched exponential tail starts later (fitted parameters: μ = 1.33, θ = 14 s, β = 0.75). For the distribution of pauses (B), the inclusion of data from the borders results in the stretched exponential tail being less pronounced such that the power-law spans over much longer time scales (fitted parameters: μ = 1.45, θ = 89.9 s, β = 0.579). (DOC) [file pcbi.1002498.s001.doc]

**
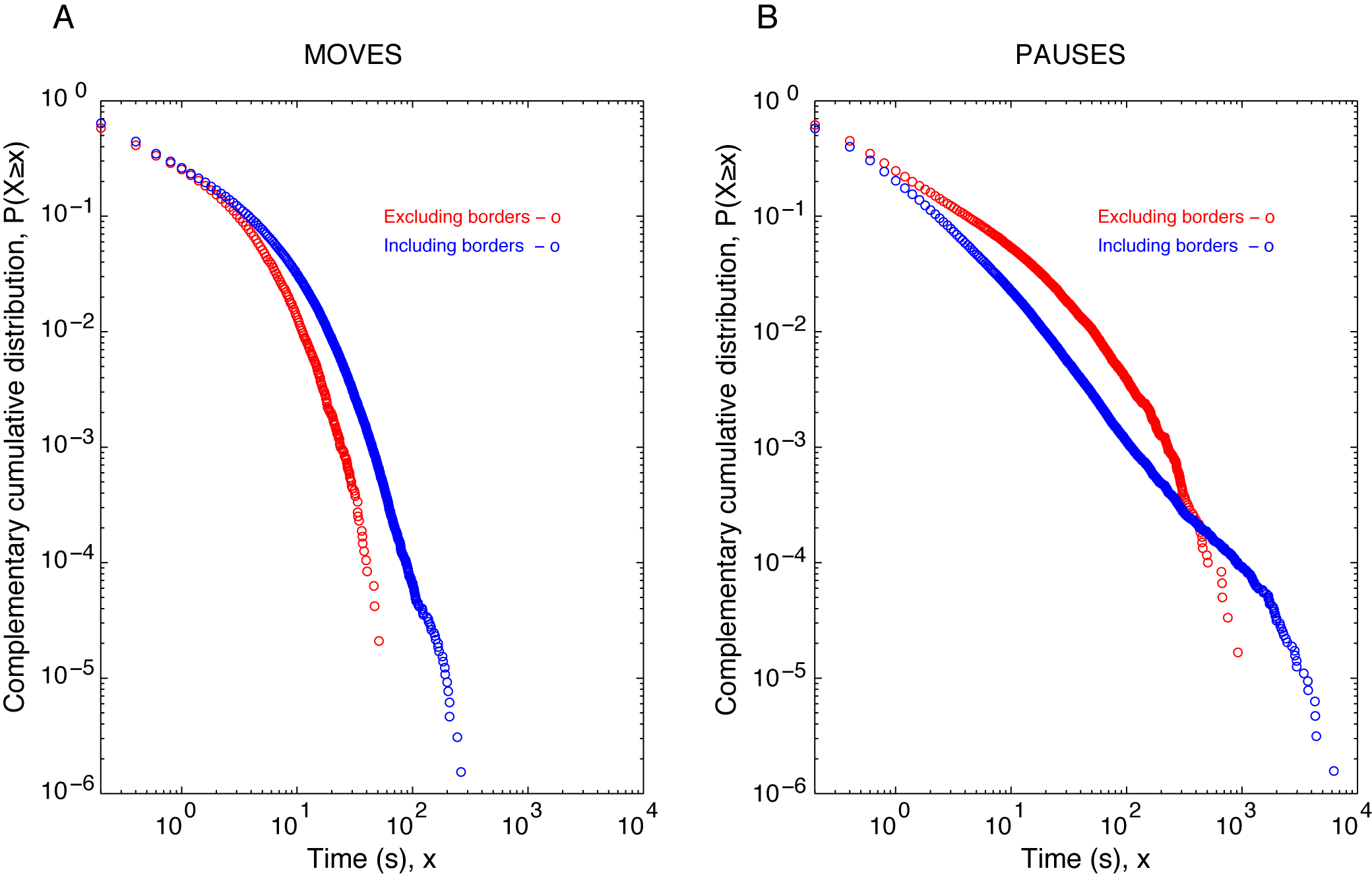
**

**Figure S1. Complementary cumulative distribution functions including and excluding boundary data**
