# Supplementary figures and images for: Intermittent Motion in Desert Locusts: Behavioural Complexity in Simple Environments
Source: PLoS Comput Biol. 2012 May 10;8(5):e1002498. doi: 10.1371/journal.pcbi.1002498 (PMC3349720; doi:10.1371/journal.pcbi.1002498)

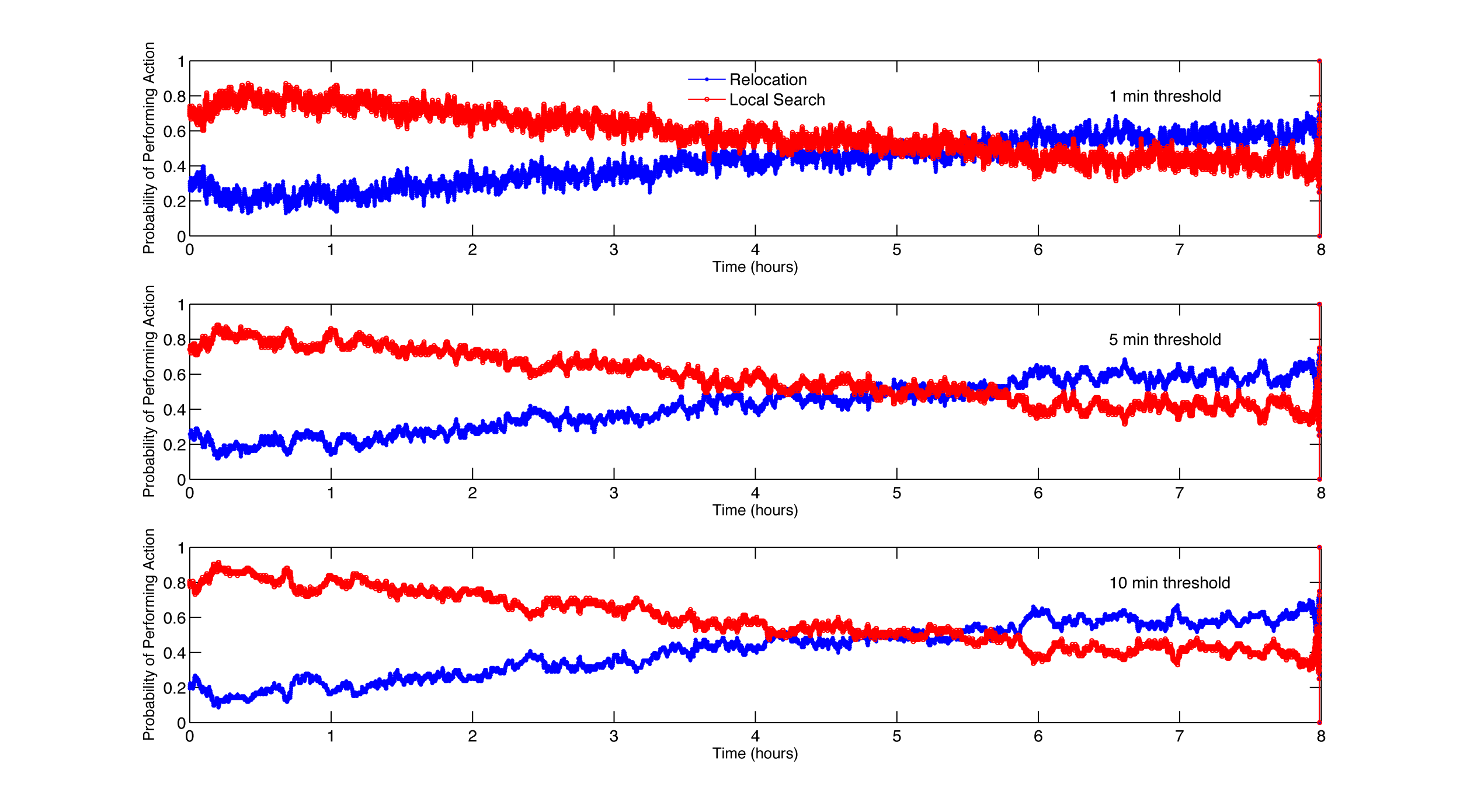


**Figure S2. Behavioural mode thresholds**

Supplement: Figure S2 — Behavioural mode thresholds. The probability of being in local search or relocation modes over time for different time duration thresholds. In the PS algorithm used to detect the two behavioural modes, the time duration threshold parameter represents the minimum time threshold for a behavioural mode to be sustained in order to be considered different from the previous mode. It is a constant numerical value that allows the allocation of breakpoints in the cumulative deviation series (Knell and Codling 2011) thereby distinguishing distinct modes, according to this minimum behavioural mode time duration threshold. Time thresholds of 1 min (top panel), 5 min (middle panel) and 10 min (bottom panel) were tested, meaning that a mode should last at least 1, 5, or 10 minutes. All thresholds tested yield the same qualitative results. (DOC) [file pcbi.1002498.s002.doc]

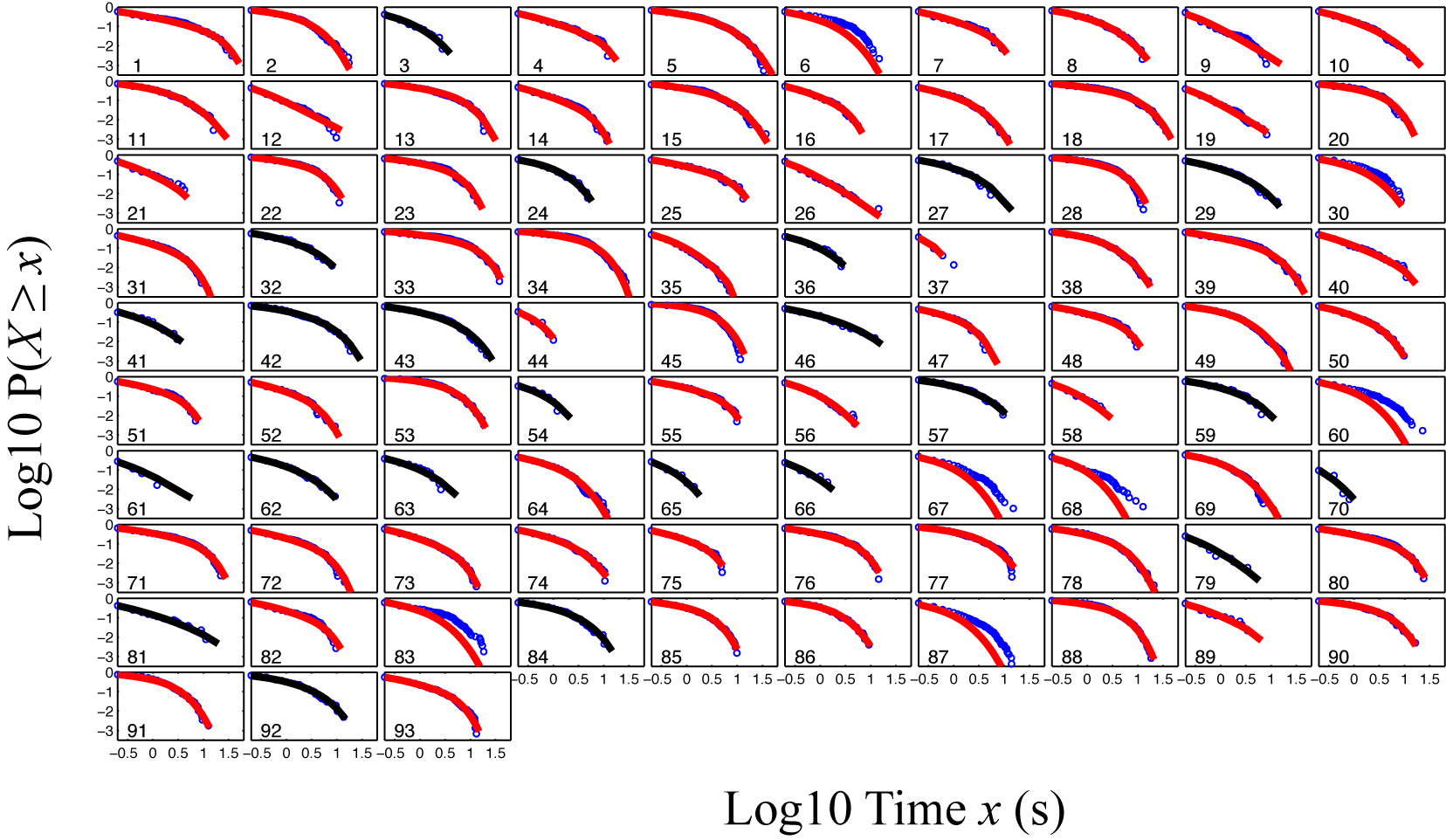


**Figure S3. Complementary cumulative distribution functions for move lengths**

Supplement: Figure S3 — Complementary cumulative distribution functions for move lengths. Each subplot shows a log-log plot of the complementary cumulative distribution function of the empirical data (blue) of different move lengths, (x) exhibited by each individual locust and the best model fit, either the exponential followed by power-law with exponential tail model fit (red) or the power-law with exponential tail model fit (black). Numbers in each subplot indicate individual number (1 to 93). (DOC) [file pcbi.1002498.s003.doc]

**
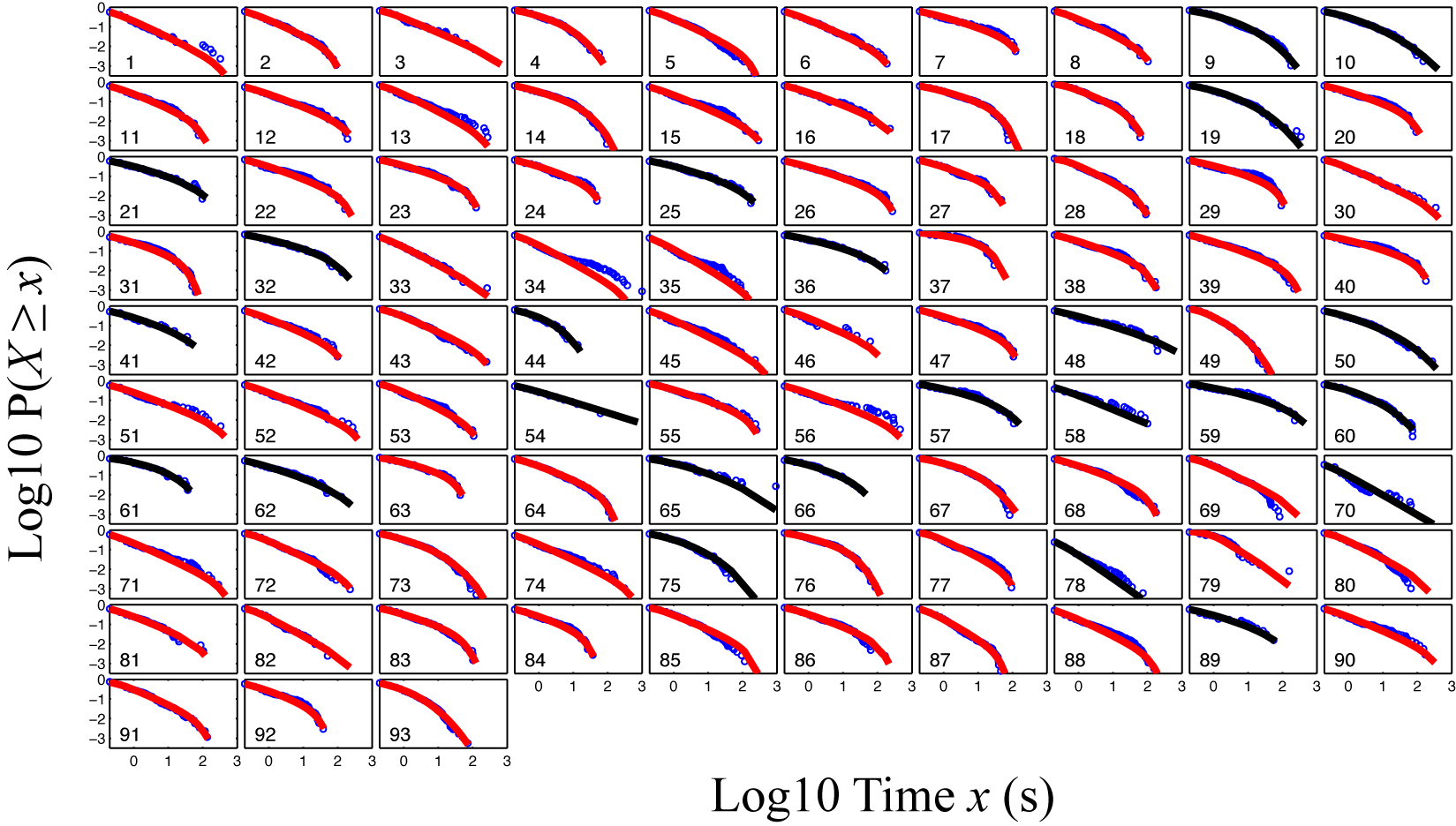
**

**Figure S4. Complementary cumulative distribution functions for pause lengths**

Supplement: Figure S4 — Complementary cumulative distribution functions for pause lengths. Each subplot shows a log-log plot of the complementary cumulative distribution function of the empirical data (blue) of different pause lengths (x) exhibited by each individual locust and the best model fit, either the exponential followed by power-law with exponential tail model fit (red) or the power-law with exponential tail model fit (black). Numbers in each subplot indicate individual number (1 to 93). (DOC) [file pcbi.1002498.s004.doc]

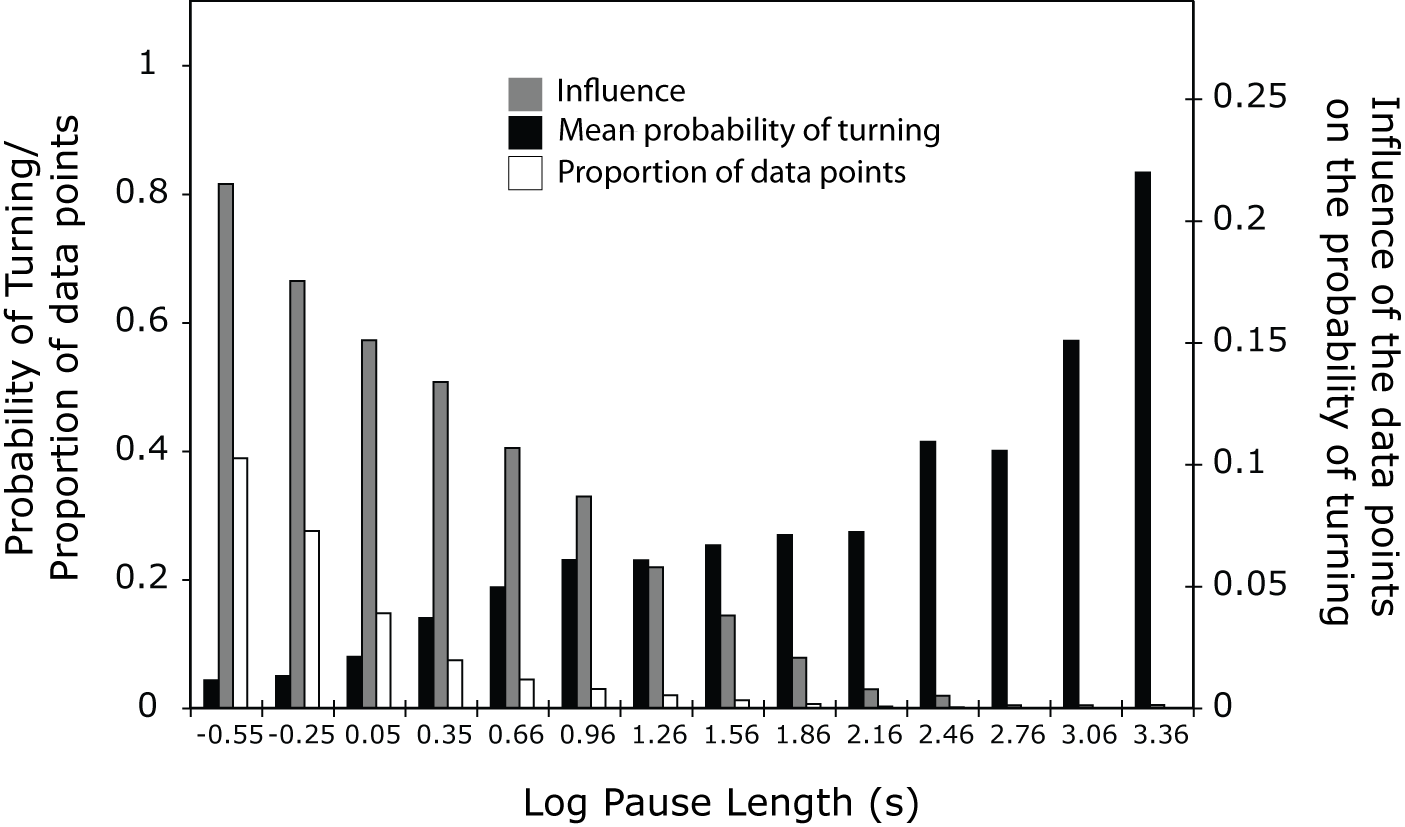


**Figure S5. The influence of pause length on the probability of turning after a pause**

Supplement: Figure S5 — The influence of pause length on the probability of turning after a pause. Bar chart showing, on the left axis, the proportion of data points (white) and the mean probability of turning after a pause (black) for each pause length bin class (log binned classes). The influence (grey) of the data points on the mean probability of turning after a pause for each pause length is also shown (right axis). This was calculated by multiplying the mean probability of turning by the proportion of data points within each pause length bin class. (DOC) [file pcbi.1002498.s005.doc]

**
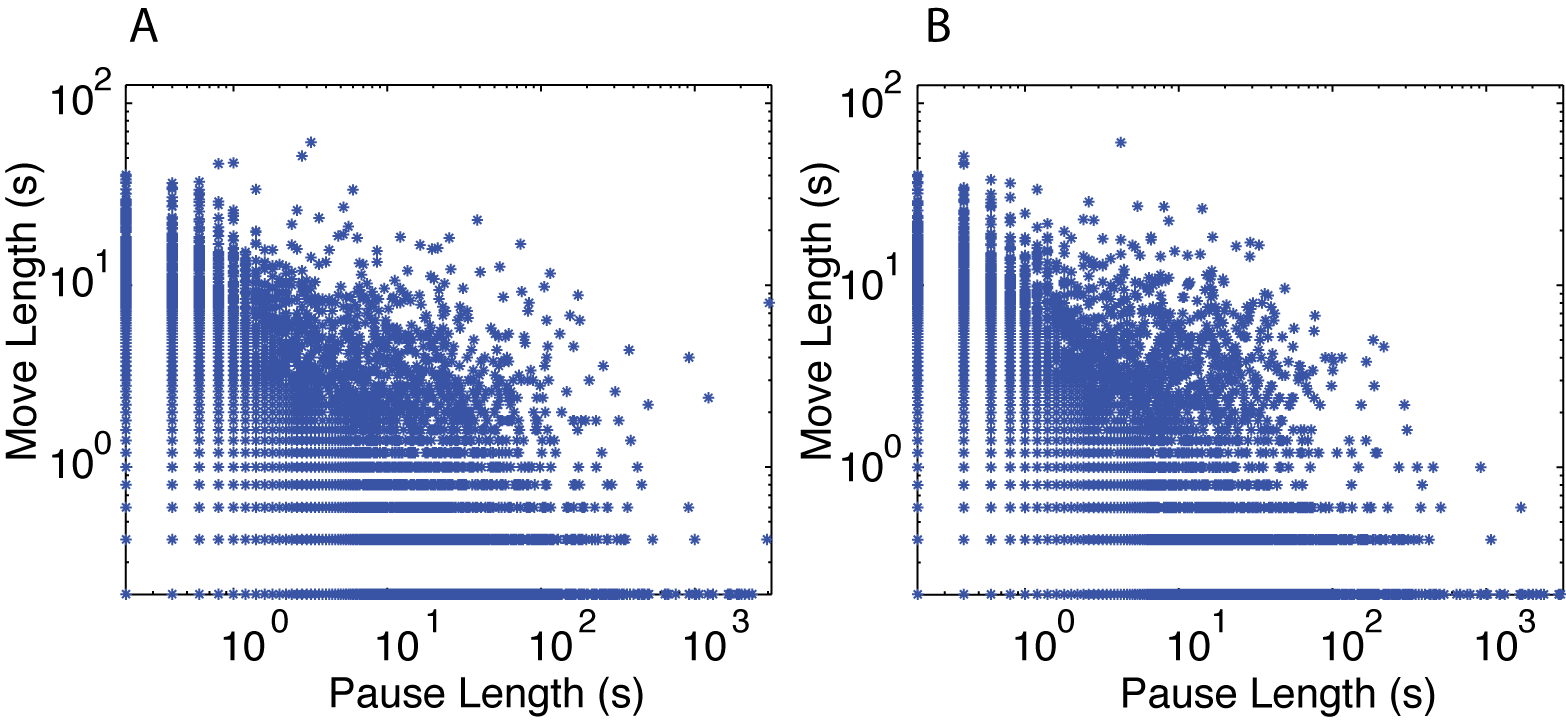
**

**Figure S6. Pause lengths and move lengths proceeding or following pauses**

Supplement: Figure S6 — Pause lengths and move lengths proceeding or following pauses. The relationship between pause length (s) and move length (s) for moves immediately preceding (A) or following (B) pauses for all locusts. The relationship between pause length and the following move length, or pause length and the preceding move length show a similar pattern (since moves after one pause are before another). (DOC) [file pcbi.1002498.s006.doc]

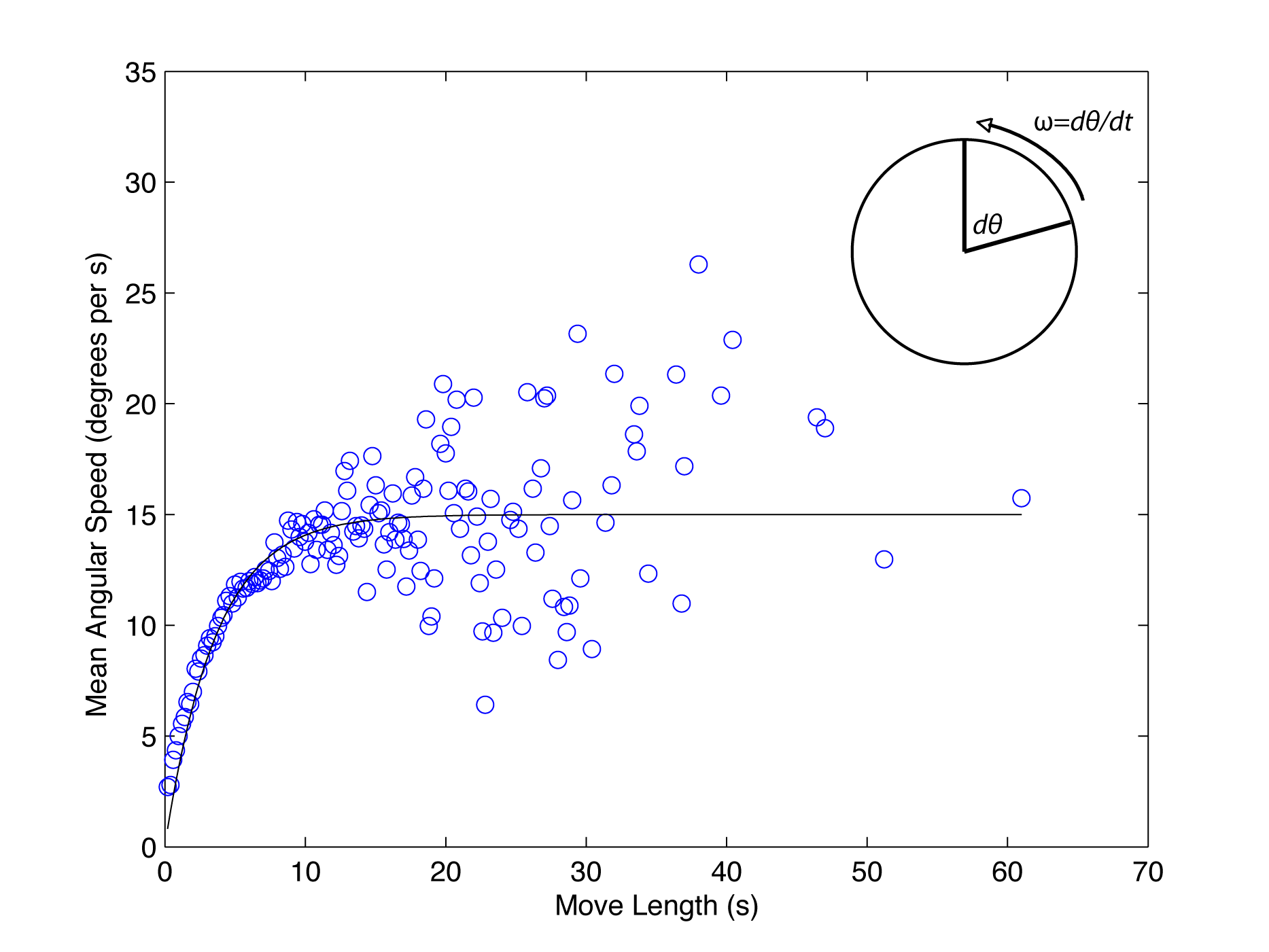


**Figure S7. Angular speed for different move lengths**

Supplement: Figure S7 — Angular speed for different move lengths. The angular speed (in degrees per s), ω, is measured as dθ/dt, where dθ is the angle (in degrees) moved between the first and last frame of the move, and dt is the move length in s. The black line shows a non-linear least squares fit (of the type: , where a = 3; c = 18, in Matlab 2010b) to the data. The mean angular speed saturates to 15 degrees/s at move lengths of 15 s. (DOC) [file pcbi.1002498.s007.doc]

**Table S1. Individual model fit results for moves**


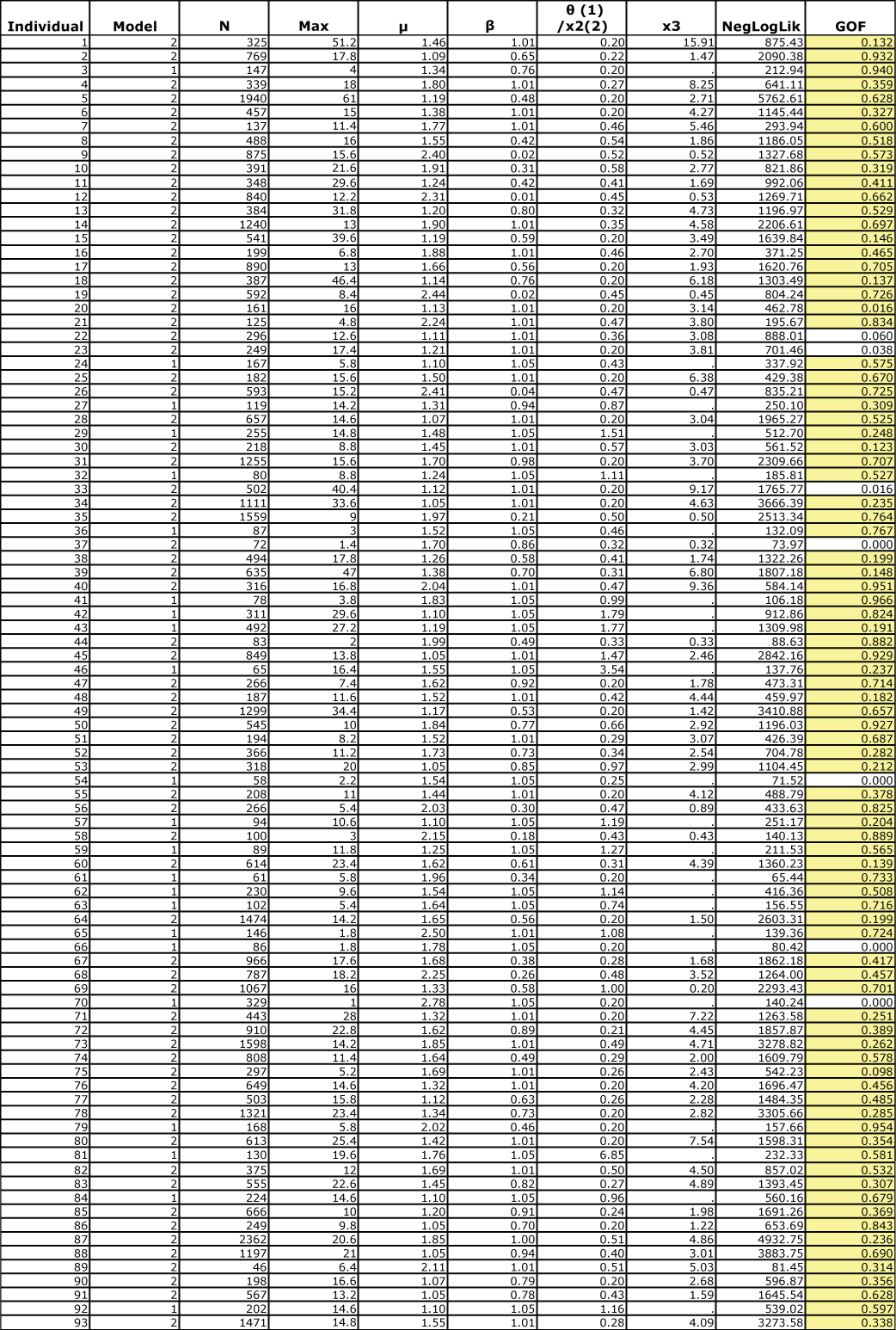

Supplement: Table S1 — Individual model fit results for moves. For the distribution of moves of each individual locust we calculated: the best fit model to the data, either model 1 or model 2, Model; the total number of move lengths, N; the maximum move length, Max (in s), for each individual (the minimum move length for all individuals is 0.2 s); the Lévy exponent, μ; the parameter that tells us the deviation of the tail from an exponential, β (where β = 1 is an exponential tail, and β = 0 is a power-law tail); for model 1 the mean lifetime (or characteristic) move length (in s), θ (1) ; for model 2 the move length value (in s) delimiting the beginning of the power law regime, x2(2); move length value (in s) delimiting the end of the power law regime, x3, (the dots in column x3 show where model 1 was a better fit than model2); the negative log-likelihood function, NegLogLik; the goodness of fit test statistic value, GOF; the p-value from a goodness-of-fit test telling us whether the model is reliable or not (from Edwards et al. (2007)), where p-values>0.1 are a good fit of the model to the data (highlighted). (DOC) [file pcbi.1002498.s008.doc]

**Table S2. Individual model fit results for pauses**


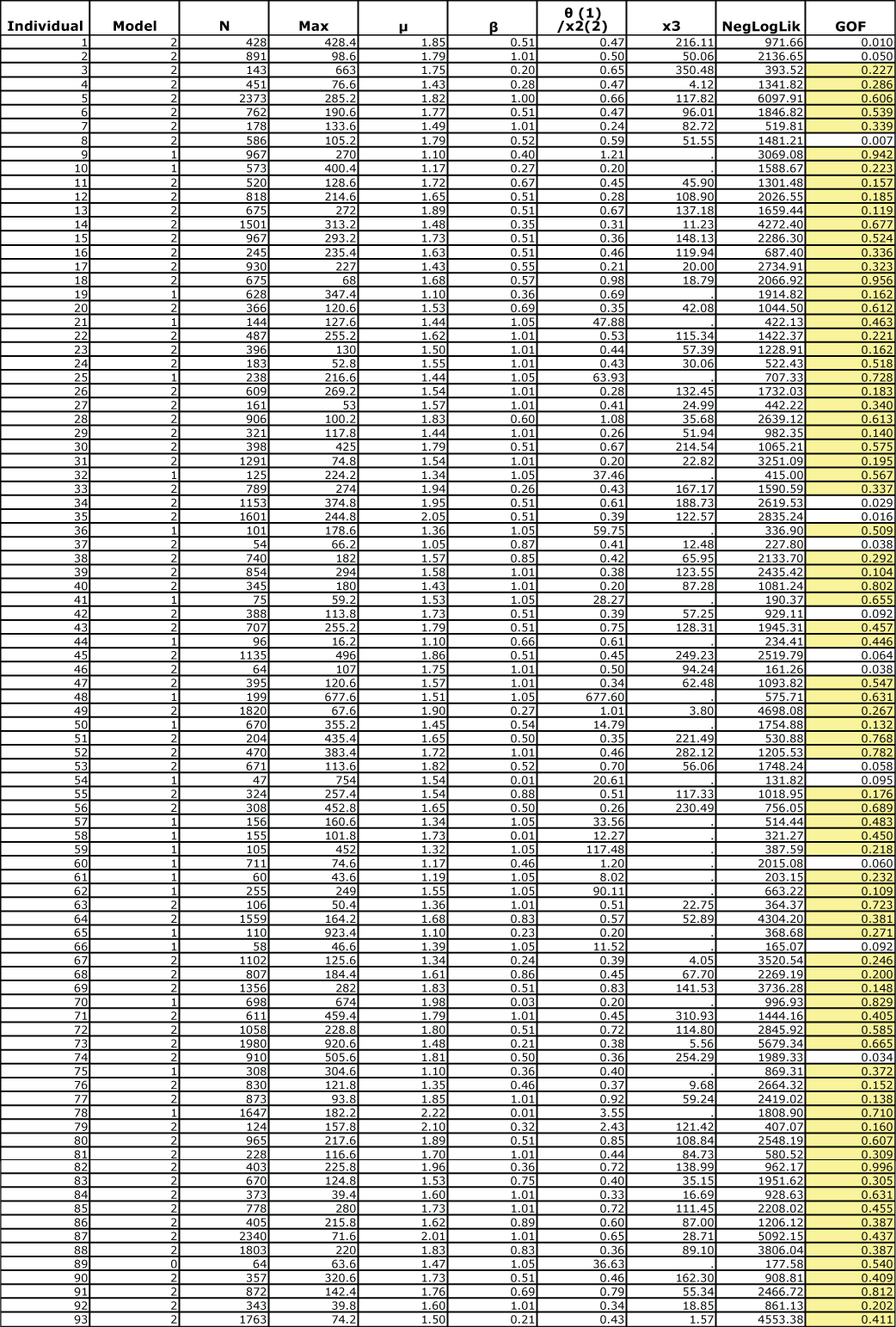

Supplement: Table S2 — Individual model fit results for pauses. For the distribution of pauses of each individual locust we calculated: the best fit model to the data, either model 1 or model 2, Model; the total number of pause lengths, N; the maximum pause length, Max (in s), for each individual (the minimum pause length for all individuals is 0.2 s); the Lévy exponent, μ; the parameter that tells us the deviation of the tail from an exponential, β (where β = 1 is an exponential tail, and β = 0 is a power-law tail); for model 1 the mean lifetime (or characteristic) pause length (in s), θ (1); for model 2 the pause length value (in s) delimiting the beginning of the power law regime, x2(2); pause length value (in s) delimiting the end of the power law regime, x3, (the dots in column x3 show where model 1 was a better fit than model2); the negative log-likelihood function, NegLogLik; the goodness of fit test statistic value, GOF; the p-value from a goodness-of-fit test telling us whether the model is reliable or not (from Edwards et al. (2007)), where p-values>0.1 are a good fit of the model to the data (highlighted). (DOC) [file pcbi.1002498.s009.doc]
