# Supplementary material for: Intermittent Motion in Desert Locusts: Behavioural Complexity in Simple Environments
Source: PLoS Comput Biol. 2012 May 10;8(5):e1002498. doi: 10.1371/journal.pcbi.1002498 (PMC3349720; doi:10.1371/journal.pcbi.1002498)
Supplement: Text S1 — The supporting text provides further details of the materials and methods used. These include correlation analyses between moves and pauses, the behavioural modes analysis, and the quantification of move speeds. In addition, the supporting text also shows the mathematical description of the probabilistic models used in SPWMC, details of individual locust data analyses, and calculations of the different models used in our investigation. (DOC) [file pcbi.1002498.s010.doc]

**Supporting Text S1**

**Materials and Methods**

**Correlation analyses for moves and pauses**

We computed the partial autocorrelation and cross-correlation functions for move and pause lengths using ‘pacf’ and ‘ccf’ functions of R (Version 2.8.0). Given a time series z*_t_*, the partial autocorrelation of lag *k* is the autocorrelation between z*_t_* and z*_t + k_* with the linear dependence of z*_t + 1_* through to z*_t + k − 1_* removed; equivalently, it is the autocorrelation between z*_t_* and z*_t − k_* that is not accounted for by lags 1 to *k*-1, inclusive. Partial correlation is useful if one expects the small-scale correlations to pervade on higher order correlations. The cross-correlation function consists of looking at the crossed correlations between two series at different scales. White noise or pure random stochastic variables show values of zero for both partial autocorrelation and cross-correlations.

**Behavioural modes analysis**

In the Partial Sums algorithm, the minimum time threshold that a behavioural mode should last in order to be considered different from the previous mode was chosen as 5 minutes. Other threshold values were also tested but did not qualitatively change the results (see Figure S2)

**Move speeds**

We quantify the move speed of each locust by measuring the angular speed (a measure of how fast the locust rotates around the arena) between the first and last frame of the move. The angular speed or frequency (in degrees per s), ω, was measured as dθ/dt, where dθ is the angle (in degrees) moved between the first and last frame of the move, and dt is the move length in s. We examined the relationship between angular speed and move length. We observed a saturation curve of angular speed with increasing move length. Thus we fitted a saturating function, using a non-linear least squares fit, in order to obtain the saturation value of velocity. However, we observed over-dispersion around this saturation value (values that were much larger and values that where much slower). We used this saturation value as the threshold to define fast and slow moves (see Figure S7), as described in the Results and Discussion.

**Mathematical description of the probabilistic models used in SPWMC**

In order to determine which probabilistic model best fits the distribution of moves and pauses for each locust, we carried out sequential pointwise model comparison (SPWMC) tests. The SPWMC analysis consisted of conducting maximum likelihood estimates and Akaike weight computations (wAIC) and the relative likelihood of each model compared with the likelihood of the best-fit model (Bartumeus *et al.* 2010). The value of the wAIC gives the weight of evidence in favour of a model, where wAIC=1 is the maximum weight of evidence. The analysis also showed whether models could fit different regimes of the data.

In our study we perform tests examining the following models:

**1. Bounded (truncated) power law**: $f(x)=Cx^{-\mu}$with $x\in[a,b]$wherein $C$is the normalization constant given by $C=(\mu-1)/(a^{1-\mu}-b^{1-\mu})$,

**2.** **Exponential**: $f(x)=Ce^{-\alpha x}$with $x\in[a,\infty]$wherein C the normalization constant given by $C=\alpha/(e^{-\alpha a})$, $\alpha$ is the scale parameter, equivalently$\lambda$ is the inverse scale parameter, that is, $\alpha=1/\lambda$.

**3. Pure power law**: $f\left(x\right)=Cx^{-\mu}$ with $x\in[a,\infty]$, where $C=\left(\mu-1\right)a^{\mu-1}$ is the normalization constant.

**Detection of power law regimes in moves and pauses: individual locust data analyses**

In order to accurately determine the value of µ of the bounded power-law distributions observed in our move length and pause length datasets, we performed fits with two general models that explicitly illustrate the fact that empirical data can only be described as the composite of different yet simple probabilistic models fitting different regimes of the data (Bartumeus and Raposo 2011). Model 1 consists on a power-law distribution with a stretched exponential distribution as the tail (i.e. large values regime) function. The stretched exponential distribution is an exponential distribution with a parameter, β (where 0<β<1), which accounts for deviations from exponential behaviour at the tail. β=1 represents pure exponential behaviour, and the smaller the β value, the fatter the tail. Model 2 consists on an exponential distribution for the small values regime, followed by a power-law with a stretched exponential distribution as the tail function (see details below, also Bartumeus and Raposo (2011)). We determined which of the two models above best fits the data for each individual.

Standard goodness-of-fit tests (G-tests or Kolmogorov-Smirnov (KS) tests) only work for simple (not noisy) data, and often fail with very large samples as they are too powerful/sensitive (Bolker 2008). If we performed these standard tests we would obtain very small p-values (< 0.1), suggesting a poor fit of the model to our data, and thus rejection of the model as a good fit. However, if we performed a Student’s t-test of the deviations between the observed (data) and the expected (model) frequencies we did not reject the null hypothesis (H_0_=deviations are zero, p-value > 0.1), suggesting our model was a good fit to the data. This result was inconsistent with the standard goodness-of-fit tests’ results. Furthermore we did not observe any systematic bias or any useful biological information in the deviations, thereby reinforcing the idea that these are good null probabilistic models to describe a locust’s movement.

We therefore modified the KS-test performed in Clauset *et al.* (2009). We reduced our sample data set (pre-binned probability density functions (PDF), see calculations below) by combining data across larger move/pause length bin classes to slightly smooth out the fluctuations between the model and the data, and also to avoid ties (repeated values) in the test. We computed a two-sample KS-test between the empirical data and the fitted model (D_0_ statistic). Then we generated random samples (N=999) based on the fitted model and compared them to the empirical data as well (D_r_; from 1…N). We computed a p-value based on the probability of finding: D_0_ < D_r_. The larger the probability, the larger the likelihood that our data came from the proposed model. Typically p-values > 0.1 indicate a good fit of the model to the data.

We also visually checked the model fits by plotting each fit together with the empirical complementary cumulative distribution function (CCDF). We computed the empirical CCDFs by plotting for a variable *x* (here either move or pause lengths in frames) the proportion of observations that were equal to or larger than *x*, i.e., *P(X≥x)* on a logarithmic scale (Sim*s et a*l. 2007).

Tables S1 and S2 show the results of our model analyses for the moves and pauses, respectively. The individual move and pause length distributions are shown together with the model fits in Figures S3 and S4, respectively. KS-test p-values of greater than 0.1 indicate a good fit of our model to the empirical data. 84 out of 93 cases have KS-test p-values greater than 0.1 for moves (Table S1), and 79 out of 93 individuals examined have KS-test p-values greater than 0.1 for pauses (Table S2).

The two fitted models capture the overall statistical signature of move and pause lengths in the data, and at the same time show enough degrees of freedom to cope with the observed individual large variation. At the individual level, the scaling exponents show variation (however all of them are in the Lévy regime (1<µ≤3)), the stretched exponential tail starts at different move/pause lengths for different individuals, and the shape of the tails can go from something exponential to fatter tails (see µ,θ, and β in Tables S1 and S2, and Figures S3 and S4). These results suggest that the distribution shows a power-law regime, but always with additional complexity, even at the individual level.

**Model 1: Probability density function and maximum likelihood calculations for a power law distribution with a stretched exponential tail**

Let us consider now the power-law probability density function (PDF) with stretched exponential cut-off without an upperbound. The PDF of interest is thus:

$p\left(x\right)=Ax^{-\mu}\exp\left[-\left(x/\theta\right)^{\beta}\right]$,$x\geq a$. (1)

The model shown in Eq. (1) has three parameters: the scaling exponent of the power-law ($\mu$), the characteristic time of the exponential ($\theta$), and a parameter accounting for deviations from exponential decay at the tail (β). All parameters must be positive, with $\mu>1$ and $0<\beta<1$. The smaller the $\beta$, the larger the deviation from exponential behavior (the "fatter" the tail).

The normalization constant *A* is such that

$\int_{a}^{\infty} p\left(x\right)dx=1$, (2)

that is:

$A\int_{a}^{\infty} x^{-\mu}\exp\left[-\left(x/\theta\right)^{\beta}\right]dx=1$ . (3)

We now make the change of variable, $y=(x/\theta)^{\beta}$, in the equation above. We note that

$dy=\frac{\beta}{\theta^{\beta}}x^{\beta-1}dx=\frac{\beta}{\theta}\left(\frac{x}{\theta}\right)^{\beta-1}dx=\frac{\beta}{\theta}y^{\left(\beta-1\right)/\beta}dx$, (4)

or

$dx=\frac{\theta}{\beta}y^{-\left(\beta-1\right)/\beta}dy=\frac{\theta}{\beta}y^{-1+1/\beta}dy$. (5)

Substituting this result into Eq. (3), we find

$A\frac{\theta}{\beta}\int_{\left(a/\theta\right)^{\beta}}^{\infty} \theta^{-\mu}y^{-\mu/\beta}y^{1/\beta-1}\exp\left(-y\right)dy=1$. (6)

Now, defining the incomplete upper Gamma function as

$\Gamma_{\mathrm{up}}\left(c;k\right)=\int_{k}^{\infty} y^{c-1}\exp\left(-y\right)dy$, (7)

we note that the integral in Eq. (6) above can be expressed as

$\int_{\left(a/\theta\right)^{\beta}}^{\infty} y^{(1-\mu)/\beta-1}\exp(-y)dy=\Gamma_{\mathrm{up}}\left(\left[1-\mu\right]/\beta;\left[a/\theta\right]^{\beta}\right)$. (8)

Finally, by substituting Eq. (8) into Eq. (6), we obtain

$A=\frac{\beta}{\theta^{1-\mu}}\left\{\Gamma_{\mathrm{up}}\left(\left[1-\mu\right]/\beta;\left[a/\theta\right]^{\beta}\right)\right\}^{-1}$. (9)

*Expressions for the analysis of pre-binned data*

Following Edwards *et al.* (2007), in order to account for the pre-binned character of our data (i.e. frames) in our maximum likelihood fitting procedure we need to define the function

*P*(being in bin $j|\lambda$) =$\int_{a+\left(j-1\right)w}^{a+jw} p\left(x\right)dx$. (10)

By inserting Eq. (1) above, we obtain

*P*(being in bin $j|\lambda$) = $A\int_{a+\left(j-1\right)w}^{a+jw} x^{-\mu}\exp\left[-\left(x/\theta\right)^{\beta}\right]dx$. (11)

We now make exactly the same change of variable as before, so to obtain

*P*(being in bin $j|\lambda$) = $A\frac{\theta^{1-\mu}}{\beta}\int_{y_{j-1}}^{y_{j}} y^{\left(1-\mu\right)/\beta-1}\exp\left(-y\right)dy$, (12)

where we define

$y_{j-1}=\left(\frac{a+\left(j-1\right)w}{\theta}\right)^{\beta}$ (13)

and

$y_{j}=\left(\frac{a+jw}{\theta}\right)^{\beta}$. (14)

Finally, by substituting above the expression for *A*, Eq. (9), we end up with

*P*(being in bin $j|\mu,\theta,\beta$) =$\frac{\Gamma_{\mathrm{up}}\left(\left[1-\mu\right]/\beta;y_{j-1}\right)-\Gamma_{\mathrm{up}}\left(\left[1-\mu\right]/\beta;y_{j}\right)}{\Gamma_{\mathrm{up}}\left(\left[1-\mu\right]/\beta;\left[a/\theta\right]^{\beta}\right)}$. (15)

The log likelihood function is then:

$\iota(\mu,\theta,\beta|data)=\sum_{j=1}^{J} d_{j}\log\left[P(beinginbinj|\mu,\theta,\beta)\right]$ (16)

where $d_{j}$is the number of counts at bin $j$. Eq. (16) can be used to find the maximum likelihood estimation of the parameters given a specific prebinned dataset.

**Model 2: Probability distribution function and maximum likelihood calculations for a model showing an exponential distribution followed by a power law distribution with a stretched exponential tail**

We perform a PDF function, *p*(*x*), with expression given by:

*A*exp(*-α x*), *x*_1_ *≤ x < x*_2_ ;

*p*(*x*) = *Bx^-μ^ , x*_2_ *≤ x < x*_3_ ;

*C*exp[*-*(*x/θ*)*^β^*)]*, x ≥ x*_3_ (17)

In Eq. (17) we have four distribution parameters (*α,μ,β,θ*), 2 breakpoint values*, x*_2_*, x*_3_, and three constants (A, B, C). Note that *x*_1_ is assumed to be the minimum value of the data set (therefore, it is not a fitting parameter). *x*_2_ and *x*_3_ are functions of the four distribution parameters (see Eq. 26 and 29) and mark the boundaries (start and end) of the power law regime. Due to the explicit relationship between the distribution parameters and the breakpoint values *x*_2_ and *x*_3_, we end up performing a four parameter fit using the following parameters: *μ,β*, *x*_2_ and *x*_3_. From these four we can automatically derive *α* and *θ*.

There are some constraints that the PDF *p*(*x*) must obey:

a) it must be normalized to the unit in the interval *x ≥ x*_1_, that is:

$\int_{x_{1}}^{\infty} p(x)dx=1$ (18)

b) it must be continuous over the entire interval, so that

*p*(*^-^*)(*x*_2_) = *p*^(+)^(*x*_2_) and *p*(*^-^*)(*x*_3_) = *p*^(+)^(*x*_3_) (19)

where, e.g., *p*(*^-^*)(*x*_2_) means the exponential part of the pdf evaluated at *x* = *x*_2_, whereas

*p*^(+)^(*x*_2_) means its power-law part also at *x* = *x*_2_.

c) it must be smooth over the entire interval, implying in

$\frac{dp^{(-)}}{dx}\left|_{x=x_{2}}\right.=\frac{dp^{(+)}}{dx}\left|_{x=x_{2}}\right.$ and $\frac{dp^{(-)}}{dx}\left|_{x=x_{3}}\right.=\frac{dp^{(+)}}{dx}\left|_{x=x_{3}}\right.$ (20)

this condition on the derivative of *p*(*x*) avoids the existence of kinks at *x* = *x*_2_ and

*x* = *x*_3_.

Notice, therefore, that Eqs. (18)-(20) furnish us with 5 equations. Since we have counted above 7 constants to be determined [the sets (*α,μ,β,θ*) and (*A, B, C*)] (or 10, depending on the interpretation of *x*_1_*, x*_2_ and *x*_3_), then, at the end, we will deal with only 2 (or 4, by also including *x*_2_ and *x*_3_) independent fitting parameters, from which the rest can be calculated.

We now start to apply Eqs. (18)-(20) to allow us to express all the parameters in terms of the independent ones. Let's begin with Eq. (19), from which we obtain:

*A*exp(*-α x*_2_) = *Bx*_2_*^-μ^* (21)

and

*Bx*_3_*^-μ^ = C*exp[*-*(*x*_3_*/θ*)*^β^*)] (22)

In order to apply Eq. (20), we first need to differentiate *p*(*x*):

*-αA*exp(*-α x*), *x*_1_ *≤ x < x*_2_ ;

$\frac{dp(x)}{dx}$ *=*-*μBx^-μ^*^-1^*, x*_2_ *≤ x < x*_3_ ;

$-\frac{\beta Cx^{\beta-1}}{\theta^{\beta}}$exp[*-*(*x/θ*)*^β^*)]*, x ≥ x*_3_ (23)

With this in mind, Eq. (20) leads to:

*-αA*exp(*-α x*_2_) = -*μBx^-μ^*^-1^ (24)

and

-*μBx*_3_*^-μ^*^-1^ = $-\frac{\beta Cx_{3}^{\beta-1}}{\theta^{\beta}}$ exp[*-*(*x*_3_*/θ*)*^β^*)] (25)

Now, before applying Eq. (18), let's manipulate a little Eqs. (21), (22), (24) and (25). By dividing Eq. (24) by Eq. (21), we obtain

$\alpha=\frac{\mu}{x_{2}}$ (26)

so that substituting back either in Eq. (21) or in Eq. (24),

*B =* $Ax_{2}^{\mu}\exp(-\mu)$ (27)

Now, by dividing Eq. (25) by Eq. (22), we obtain

$\left(\frac{x_{3}}{\theta}\right)^{\beta}=\frac{\mu}{\beta}$ (28)

or

$\theta=x_{3}\left(\frac{\beta}{\mu}\right)^{1/\beta}$ (29)

Substituting back either in Eq. (22) or in Eq. (25), and with the help of Eq. (26), we obtain

$C=A\left(\frac{x_{2}}{x_{3}}\right)^{\mu}\exp\left[\mu(-1+1/\beta)\right]$ (30)

We now apply the normalization condition. By inserting Eq. (17) into Eq. (18), we obtain

$A\int_{x_{1}}^{x_{2}} \exp(-\alpha x)dx+B\int_{x_{2}}^{x_{3}} x^{-\mu}dx+C\int_{x_{3}}^{\infty} \exp\left[-(x/\theta)^{\beta}\right]dx=1$ (31)

The first and second integrals above are relatively easy to solve. The third one needs the change of variable that we usually make: *y* = (*x/θ*)*^β^*, so that

$dx=\frac{\theta}{\beta}y^{-1+1/\beta}dy$ (32)

Therefore, after solving all integrals in Eq. (31), we get

$-\frac{A}{\alpha}\left[\exp\left(-\alpha x_{2}\right)-\exp\left(-\alpha x_{1}\right)\right]+\frac{B}{\left(1-\mu\right)}\left(x_{3}^{1-\mu}-x_{2}^{1-\mu}\right)+\frac{\theta C}{\beta}\Gamma_{\mathrm{up}}\left(1/\beta;\left(x_{3}/\theta\right)^{\beta}\right)=1$ (33)

where the incomplete upper gamma function is usually defined as:

$\Gamma_{\mathrm{up}}\left(c;k\right)=\int_{k}^{\infty} y^{c-1}\exp\left(-y\right)dy$ (34)

Finally, by substituting Eqs. (26)-(30) into Eq. (33), and some algebraic manipulation, we obtain

$$A=\frac{\mu\exp(\mu)}{x_{2}}\left\{-1+\exp\left[\mu\left(1-x_{1}/x_{2}\right)\right]\right.+\frac{\mu}{\left(1-\mu\right)}\left[\left(\frac{x_{3}}{x_{2}}\right)^{1-\mu}-1\right]$$

$+\left(\frac{x_{3}}{x_{2}}\right)^{1-\mu}\left(\frac{\beta}{\mu}\right)^{1/\beta-1}\left.\exp\left(\mu/\beta\right)\Gamma_{\mathrm{up}}\left(1/\beta;\mu/\beta\right)\right\}^{-1}$ (35)

Notice, above, that the coefficient *A* is simply a function of the fitting parameters *μ* and *β* (and, of course, also a function of *x*_1_*, x*_2_ and *x*_3_, as discussed). The same is true for the coefficients *B* and *C*, determined from *A* through Eqs. (27) and (30), respectively.

*Complementary cumulative distribution function calculations*

The complementary cumulative distribution function (CCDF) is generally defined as follows:

*P*(*x*) = $\int_{x_{1}}^{x} p\left(x'\right)dx'$ (36)

Since the pdf *p*(*x*) is defined in intervals, so is the cdf *P*(*x*).

- *First interval*: *x*_1_ *≤ x < x*_2_.

By inserting Eq. (17) into Eq. (36), in the proper interval, thus

*P*(*x*) = $\int_{x_{1}}^{x} A\exp\left(-\alpha x'\right)dx'$ (37)

The integral leads to

*P*(*x*) = $-\frac{A}{\alpha}\left[\exp(-\alpha x)-\exp(-\alpha x_{1})\right]$ (38)

or, by using Eq. (26),

*P*(*x*) = $-\frac{Ax_{2}}{\mu}\left[\exp\left(-\mu x/x_{2}\right)-\exp\left(-\mu x_{1}/x_{2}\right)\right]$ , *x*_1_ *≤ x < x*_2_ (39)

with *A* given by Eq. (35).

- *Second interval*: *x*_2_ *≤ x < x*_3_

The integral now reads:

*P*(*x*) = $\int_{x_{1}}^{x_{2}} A\exp\left(-\alpha x'\right)dx'+\int_{x_{2}}^{x} B\left(x'\right)^{-\mu}dx'$ (40)

Therefore,

*P*(*x*) = $\bar{A}+\frac{B}{\left(1-\mu\right)}\left(x^{1-\mu}-x_{2}^{1-\mu}\right)$ , *x*_2_ *≤ x < x*_3_ (41)

with *B* given by Eq. (27), and the new auxiliary constant defined as

$\bar{A}=\frac{Ax_{2}}{\mu}\left[\exp\left(-\mu\right)-\exp\left(-\mu x_{1}/x_{2}\right)\right]$ (42)

- *Third interval*: *x ≥ x*_3_

The respective integral is:

*P*(*x*) = $\int_{x_{1}}^{x_{2}} A\exp\left(-\alpha x'\right)dx'+\int_{x_{2}}^{x_{3}} B\left(x'\right)^{-\mu}dx'+\int_{x_{3}}^{x} C\exp\left[-\left(x'/\theta\right)^{\beta}\right]dx'$ (43)

Thus,

*P*(*x*) = $\bar{A}+\bar{B}+\frac{Cx_{3}}{\beta}\left(\frac{\beta}{\mu}\right)^{1/\beta}\left[\Gamma\mathrm{up}\left(1/\beta;\mu/\beta\right)-\Gamma\mathrm{up}\left(1/\beta;\left(\mu/\beta\right)\left(x/x_{3}\right)^{\beta}\right)\right]$ , *x ≥ x*_3_ (44)

where *C* is given by Eq. (30), we have also used Eq. (29), and the second auxiliary constant is defined:

$\bar{B}=\frac{B}{\left(1-\mu\right)}\left(x_{3}^{1-\mu}-x_{2}^{1-\mu}\right)$ (45)

*Expressions for the analysis of pre-binned data*

We start by defining the following two quantities:

*x_j_ -* _1_ = *x*_1_ + (*j -* 1)*w*  (46)

and

*x_j_* = *x*_1_ + *jw* (47)

We notice that *w* is the length of the (small) interval between two consecutive discrete values of *x_j_* , i.e.,

*x_j_* = *x_j_* _- 1_ + *w* (48)

In addition, we also define

*P*(being in bin *j* | *λ*) = $\int_{x_{j-1}}^{x_{j}} p(x)dx$ (49)

The point here is, therefore, to insert *p*(*x*) above correctly, according to the intervals defined in Eq. (17). The actual calculations of the function *P*(being in bin *j* | *λ*) are in fact very similar to those of the CCDF *P*(*x*). Indeed, we can transform the above integral into two integrals:

*P*(being in bin *j* | *λ*) = $\int_{x_{1}}^{x_{j}} p(x)dx-\int_{x_{1}}^{x_{j-1}} p(x)dx$ (50)

which, by the use of Eq. (36) can be written in terms of the usual CCDF *P*(*x*) as

*P*(being in bin *j* | *λ*) = *P*(*x_j_*) - *P*(*x_j_* _-1_) (51)

- *First interval*: *x*_1_ *≤ x < x*_2_

If both *x_j_* _-1_ and *x_j_* fall into this interval, then *x*_1_ *≤ x_j_* _-1_ and *x_j_ < x*_2_, which, by using Eq. (48), corresponds to writing *x*_1_ *≤ x_j-1_ < x*_2_ – *w.* In this case, both CCDF's in Eq. (51) are given by Eq. (39):

*P*(being in bin *j* | *λ*) = $-\frac{Ax{}_{2}}{\mu}\left[\exp\left(-\mu x_{j}/x_{2}\right)-\exp\left(-\mu x_{j-1}/x_{2}\right)\right]$, *x*_1_ *≤ x_j-1_ < x*_2_ – *w* (52)

However, in the borderline between the first and second intervals, a subtle (and rare) situation may happen: *x_j-1_* still belonging to the first interval, whereas *x_j_* is already placed in the second interval. Mathematically, the condition for such situation is

*x_j-1_x*_2_ and *x_j_* ≥ *x*_2_, or, equivalently, *x*_2_ *- w ≤ x_j-1_ < x*_2_, where we have also made use of Eq. (48). The associate function in this small borderline interval is thus:

*P*(being in bin *j* | *λ*) = $-\frac{Ax{}_{2}}{\mu}\left[\exp\left(-\mu x_{j-1}/x_{2}\right)-\exp\left(-\mu x_{1}/x_{2}\right)\right]$

$+\bar{A}+\frac{B}{\left(1-\mu\right)}\left(x_{j}^{1-\mu}-x_{2}^{1-\mu}\right)$, *x*_2_ *- w ≤ x_j-1_ < x*_2_ (53)

where, according to Eq. (51), we have considered *P*(*x_j-_*_1_) as in Eqs. (39),and *P*(*x_j_*) as in

Eq. (41).

- *Second interval*: *x*_2_ *≤ x < x*_3_

If both *x_j-_*_1_ and *x_j_* fall into this interval, then *x*_2_ *≤ x_j-1_* and *x_j_ < x*_3_, which, by using Eq. (48), corresponds to *x*_2_ *≤ x_j-1_ < x*_3_ - *w.* Therefore, both CCDF's in Eq. (51) are given by Eq. (41), so that:

*P*(being in bin *j* | *λ*) = $\frac{B}{\left(1-\mu\right)}\left(x_{j}^{1-\mu}-x_{j-1}^{1-\mu}\right)$, *x*_2_ *≤ x_j-1_ < x*_3_ – *w*  (54)

However, as in the previous case, in the borderline between the second and third intervals, *x_j-1_ < x*_3_ and *x_j_* ≥ *x*_3_, or, equivalently, *x*_3_ - *w ≤ x_j-1_ < x*_3_, where we have made use of Eq. (48). The associate function in this small borderline interval is thus:

*P*(being in bin *j* | *λ*) = $\frac{Cx_{3}}{\beta}\left(\frac{\beta}{\mu}\right)^{1/\beta}\left[\Gamma_{\mathrm{up}}\left(1/\beta;\mu/\beta\right)-\Gamma_{\mathrm{up}}\left(1/\beta;\left(\mu/\beta\right)\left(x_{j}/x_{3}\right)^{\beta}\right)\right]$

$+\bar{B}-\frac{B}{\left(1-\mu\right)}\left(x_{j-1}^{1-\mu}-x_{2}^{1-\mu}\right)$ , *x*_3_ *– w ≤ x_j-1_ < x*_3_ (55)

where, according to Eq. (51), we have considered *P*(*x_j_*_-1_) as in Eqs. (41), and *P*(*x_j_*) as in Eq. (44).

- *Third interval*: *x* ≥ *x*_3_

Finally, when both *x_j_*_-1_ and *x_j_* fall into this interval, then simply *x_j-1_* ≥ *x*_3_. In this case, we have

*P*(being in bin *j* | *λ*) =

$\frac{Cx_{3}}{\beta}\left(\frac{\beta}{\mu}\right)^{1/\beta}\left[-\Gamma_{\mathrm{up}}\left(1/\beta;\left(\mu/\beta\right)\left(x_{j}/x_{3}\right)^{\beta}\right)+\Gamma_{\mathrm{up}}\left(1/\beta;\left(\mu/\beta\right)\left(x_{j-1}/x_{3}\right)^{\beta}\right)\right]$, *x_j_*_-1_  *x*_3_ (56)

where, according to Eq. (51), we have considered both *P*(*x_j_*_-1_) and *P*(*x_j_*) as in Eq. (44).

(A note of caution: in order for the borderline calculations to work out, it is necessary to assume that *x*_2_  *x*_1_ + *w* and *x*_3_  *x*_2_ + *w*. In other words, we have to be careful and avoid simply assuming, as we did as in some tests, that *x*_2_ = *x*_1_ or *x*_3_ = *x*_2_).

In summary, when considering pre-binned data, the analysis incorporates the usual 3 intervals plus 2 borderline ranges (between the first and second, and between the second and third intervals). In the end, the expressions to be considered are Eqs. (52)-(56).

**References**

Bartumeus F, Giuggioli L, Louzao M, Bretagnolle V, Oro D, Levin SA (2010) Fishery Discards Impacts on Seabird Movement Patterns at Regional Scales. Current Biology 20:1-8.

Bartumeus, F., and E. P. Raposo. 2011. Fitting power law regimes to movement data by using composite probability density functions. In prep.

Bolker, B. M. 2008, Ecological Models and Data in R. Princeton, New Jersey, USA, Princeton University Press.

Clauset, A., C. R. Shalizi, and M. E. J. Newman. 2009. Power-law distributions in empirical data. SIAM Review 51:661-703.

Edwards, A. M., R. A. Phillips, N. W. Watkins, M. P. Freeman, E. J. Murphy, V. Afanasyev, S. V. Buldyrev et al. 2007. Revisiting Lévy flight search patterns of wandering albatrosses, bumblebees and deer. Nature 449:1044-1048.

Knell A.S. and E.A. Codling 2011. Classifying area-restricted search (ARS) using

a partial sum approach. Theoretical Ecology DOI 10.1007/s12080-011-0130-4.

Sims, D. W., D. Righton, and J. W. Pitchford. 2007. Minimizing errors in identifying Lévy flight behaviour of organisms. Journal of Animal Ecology 76:222-229.
